# A compendium and comparative epigenomics analysis of *cis*-regulatory elements in the pig genome

**DOI:** 10.1038/s41467-021-22448-x

**Published:** 2021-04-13

**Authors:** Yunxia Zhao, Ye Hou, Yueyuan Xu, Yu Luan, Huanhuan Zhou, Xiaolong Qi, Mingyang Hu, Daoyuan Wang, Zhangxu Wang, Yuhua Fu, Jingjin Li, Saixian Zhang, Jianhai Chen, Jianlin Han, Xinyun Li, Shuhong Zhao

**Affiliations:** 1 Key Lab of Agricultural Animal Genetics, Breeding and Reproduction of Ministry of Education and Key Laboratory of Swine Genetics and Breeding of Ministry of Agriculture, College of Animal Science and Technology, Huazhong Agricultural University, Wuhan, China; 2 CAAS-ILRI Joint Laboratory on Livestock and Forage Genetic Resources, Institute of Animal Science, Chinese Academy of Agricultural Sciences (CAAS), Beijing, China; 3 International Livestock Research Institute (ILRI), Nairobi, Kenya; 4 The Cooperative Innovation Center for Sustainable Pig Production, Wuhan, China

**Keywords:** Epigenomics, Functional genomics

## Abstract

Although major advances in genomics have initiated an exciting new era of research, a lack of information regarding *cis*-regulatory elements has limited the genetic improvement or manipulation of pigs as a meat source and biomedical model. Here, we systematically characterize *cis*-regulatory elements and their functions in 12 diverse tissues from four pig breeds by adopting similar strategies as the ENCODE and Roadmap Epigenomics projects, which include RNA-seq, ATAC-seq, and ChIP-seq. In total, we generate 199 datasets and identify more than 220,000 *cis*-regulatory elements in the pig genome. Surprisingly, we find higher conservation of *cis*-regulatory elements between human and pig genomes than those between human and mouse genomes. Furthermore, the differences of topologically associating domains between the pig and human genomes are associated with morphological evolution of the head and face. Beyond generating a major new benchmark resource for pig epigenetics, our study provides basic comparative epigenetic data relevant to using pigs as models in human biomedical research.

## Abstract (web summary)

To date, little is known about the regulatory landscape of the pig genome. Here, the authors characterize *cis*-regulatory elements in the pig genome using RNA-seq, ChIP-Seq and ATAC-seq, finding a higher degree of usage conservation between pig and human than mouse and human.

## Introduction

The Encyclopedia of DNA Elements (ENCODE) project, which aims to systematically improve the understanding of gene regulation, chromatin accessibility, histone modification, and chromatin structure, has examined humans^1^, as well as classic model organisms including *Caenorhabditis elegans*^2^, *Drosophila melanogaster*^3^, and mouse^4,5^. Moreover, the NIH Roadmap Epigenomics Consortium has comprehensively collected human epigenomes for tissues, providing a resource for interpreting the molecular mechanisms of human biology and diseases^6^. However, the continuation of ENCODE and Roadmap Epigenomics projects had not extended to the domestic pig (*Sus scrofa*), which not only serves as a major meat source worldwide but is also frequently used as an animal model for human diseases and as a donor for xenotransplantation^7,8^.

In recent years, transgenic pig models have become available for research on human diseases such as Huntington’s disease^7^, human type II diabetes^9^, Duchenne muscular dystrophy^10^, and Hutchinson–Gilford Progeria Syndrome^10^. The pig also represents an attractive model for the study of human breast cancer^11^ and lung cancer^12^. Moreover, xenotransplantation using pigs as donors has advanced rapidly in recent years^8,13–15^, as evidenced by the success of porcine-to-baboon heart transplantation, in which genetically modified pig hearts can function for up to 195 days^8^. Furthermore, the overall medical importance of pigs necessitates the exploration of its genomics and epigenomics to aid our understanding of human diseases and the development of new therapies.

On top of the immeasurable medical potential of this species, commercial pig farming is of great economic importance, which is reflected in a long breeding history. Large White (LW) and Duroc pigs, which are commonly used Western commercial breeds, have been subjected to intense selection for major economic traits including higher growth rates, muscle mass, and feed efficiency^16,17^. On the contrary, the Chinese local pig breeds like Meishan (MS) and Enshi Black (ES) possess relatively poor performance for these traits^17,18^, but comparatively stronger tolerance to roughage and high intramuscular fat^17,19^. Previous studies have shown that Western commercial breeds and Chinese local breeds differ in their genome-wide genetic variant landscapes^20,21^ and gene expression profiles in various tissues (e.g., subcutaneous adipose and skeletal muscle)^22,23^. To date, the determination of genotype-phenotype relationships has been limited by using only genomic sequence or gene expression profiling. Thus, the characterization of differences among *cis-*regulatory elements in diverse tissues between different pig breeds represents a timely and relevant scientific need.

In this work, we follow the guidelines of the previous ENCODE and Roadmap Epigenomics projects^1,5,6^ and design RNA sequencing (RNA-seq), chromatin immunoprecipitation followed by sequencing (ChIP-seq), an assay for transposase-accessible chromatin using sequencing (ATAC-seq), and high-throughput chromosome conformation capture (Hi-C) methodologies to identify and characterize the function of *cis-*regulatory elements in the pig genome. Here, our study fundamentally enriches *cis*-regulatory element annotation in the pig genome, which also lays a foundation for pig and human biology studies and, especially, extends ENCODE and Roadmap Epigenomics projects in the research field of large animals.

## Results

### Overview of *cis-*regulatory elements and 3D genome architecture of the pig genome

**Fig. 1:**
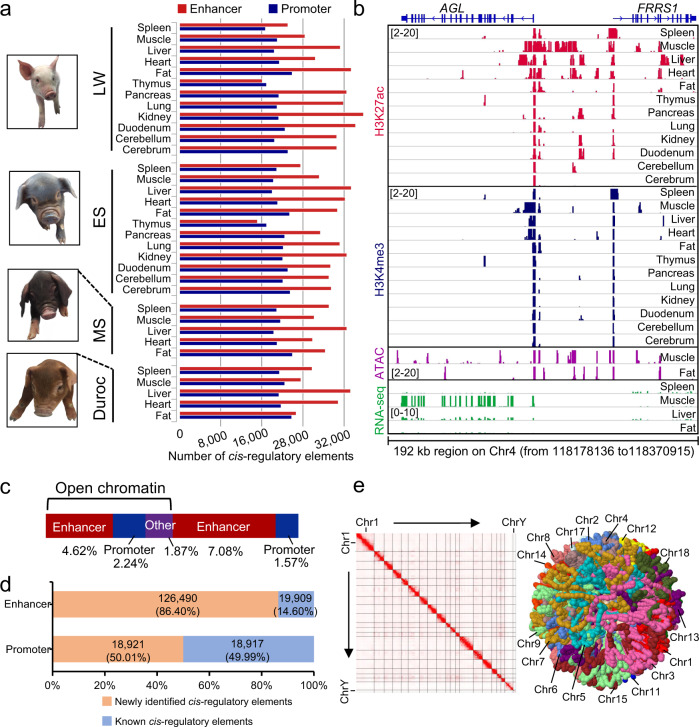
*Cis*-regulatory element landscape of the pig genome. LW, ES, and MS represent Large White, Enshi Black, and Meishan breeds, respectively. **a** Summary of *cis-*regulatory elements (enhancers and promoters) identified in various tissues of four pig breeds. **b** Genome browser views of ChIP-seq, ATAC-seq, and RNA-seq data at the *AGL* and *FRRS1* loci in tissues of LW pig. The numbers in the brackets located in the respective ChIP-seq, ATAC-seq, and RNA-seq tracks indicate their signal intensities. **c** A bar plot showing the percentage of *cis-*regulatory elements annotated in the pig genome. The percentage of enhancers (or promoters) in the black bracket were in genomic regions of open chromatin. “Other” indicates the proportion of open chromatin regions that did not overlap with enhancers or promoters. **d** Percentages of *cis-*regulatory elements newly identified in this study (salmon) and recovered by UCSC TSSs or published data (blue) from pig pluripotent stem cells^24^ and liver tissue^25^. **e** Overview of Hi-C heatmap matrix (left) and a simulated structure of 3D chromatin (right) in pig skeletal muscle. A Lorentzian objective method^27^ and GenomeFlow v2.0^28^ were used for modeling.

In this study, we generated a new benchmark resource of transcriptomics and epigenomics data for domestic pigs, comprising a total of 61 samples of 12 tissues from two-week-old piglets of two Western commercials (LW and Duroc) and two Chinese locals (MS and ES) breeds (Fig. 1a). We identified the genomic localization of histone H3 lysine 4 trimethylation (H3K4me3) and H3 lysine 27 acetylation (H3K27ac) by ChIP-seq and also characterized open chromatin regions using ATAC-seq methods (Supplementary Tables 1–3 and Supplementary Data 1–2). The saturation plots of both ChIP-seq and ATAC-seq data from different samples indicated that their sequencing depths were saturated (Supplementary Fig. 1a–b) or nearly saturated (Supplementary Fig. 1c). Furthermore, all ATAC-seq samples had over 76 million effective reads (after removing low MAPQ reads, unmapped reads, mate unmapped reads, not primary alignments, reads failing platform, duplicates, and mitochondrial reads; Supplementary Table 1), which surpassed the ENCODE project standard of 50 million effective reads for ATAC-seq data (https://www.encodeproject.org/atac-seq/). In addition to the values of cross-correlation, the fraction of reads in peaks (FRiP), transcription start site (TSS) enrichment analyses, and Principal Component Analysis (PCA), showed that our ChIP-seq and ATAC-seq data were of sufficient quality for further analyses (Supplementary Fig. 1d–i, Supplementary Tables 2–3, and Supplementary Data 2).

The datasets, which passed quality control procedures, were used to identify *cis*-regulatory sequences following the ENCODE guidelines (https://www.encodeproject.org/). In total, 220,723 non-redundant *cis-*regulatory sequences were identified, including 37,838 putative promoters, 146,399 potential enhancers, and 137,838 open chromatin regions mapped to the susScr11 genome assembly (Fig. 1a and Supplementary Fig. 2a). *AGL* and *FRRS1* genes located on pig Chr4 and *MYOG* on Chr9 were chosen as examples to illustrate the signal distributions of ChIP-seq and ATAC-seq around their TSSs along with their transcriptional expression (Fig. 1b and Supplementary Fig. 2b). Note that similar numbers of these *cis-*regulatory element sequences were also detected based on susScr3 genome assembly (Supplementary Table 4). The combined length of these non-redundant *cis-*regulatory sequences was approximately 434.92 million base pairs, accounting for 17.38% of the susScr11 genome assembly (Fig. 1c).

To assess the mapping accuracy of the above-identified *cis-*regulatory sequences, putative enhancers and promoters were compared with TSSs annotated by the University of California, Santa Cruz (UCSC) pig project and previously published ChIP-seq data (Supplementary Table 5) from pig pluripotent stem cells^24^ and liver tissue^25^. The results showed that approximately 50% of our putative promoters overlapped with promoters identified from published data or coincided with TSSs (Fig. 1d). Moreover, over 65% of the enhancers and known promoters identified from previously published studies can be recovered in our data (Supplementary Fig. 2c). Specifically, approximately 74% of enhancers and 98% of promoters from liver tissues^25^ were also identified in our liver tissue data (Supplementary Fig. 2d). These results indicate the high sensitivity of the methods used in this study. Notably, in liver tissue, over 53% and 36% of enhancers and promoters, respectively, were newly identified by this study (Supplementary Fig. 2e). Furthermore, in total, more than 86% of the enhancers and 50% of the promoters we identified have not been previously reported in the pig genome (Fig. 1d).

The 3D structure of the pig genome was assessed using in-situ Hi-C data where the skeletal muscle of one LW pig was used as a representative tissue. In total, 1,189,583,975 paired-end reads were sequenced, yielding more than 21× coverage of the pig genome. After filtering potentially artificial reads using the HiC-Pro pipeline^26^, 408,546,465 unique valid contacts were obtained, among which 290,325,259 were *cis-*contacts. Based on these contact data, chromatin conformation was plotted as the chromatin interaction frequency (Fig. 1e, left). Moreover, a 3D genome structural modeling method^27,28^ using these data clearly showed the spatial relationships between pig genomic regions (Fig. 1e, right).

### The transcriptome of the pig genome

**Fig. 2:**
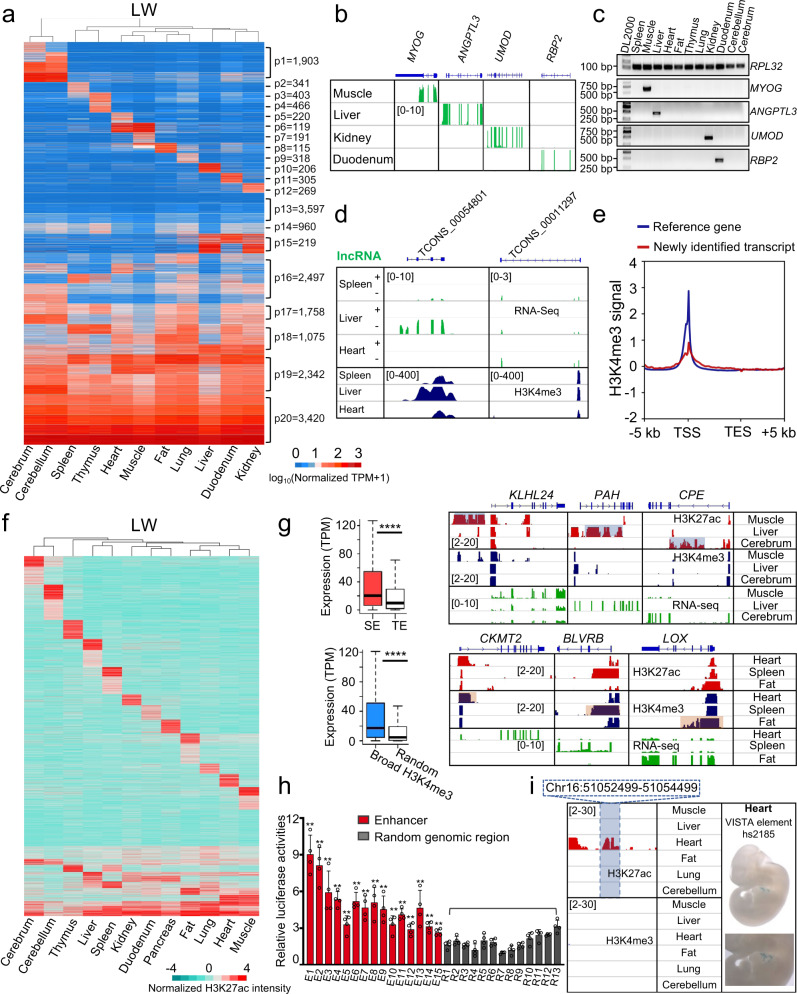
Transcriptional profiling and *cis*-regulatory elements analysis. **a** Heatmap showing gene expression patterns in 11 tissues of two-week-old Large White (LW) pigs. **b** Examples of genes exhibiting tissue-specific expression. **c** RT-PCR validation in 11 tissues of tissue-specific expression of the genes in **b**. The validation was repeated twice independently with similar results and *RPL32* was used as a control. The PCR target fragment size of *RPL32* is 93 bp, *MYOG* specifically expressed in muscle is 612 bp, *ANGPTL3* specifically expressed in the liver is 420 bp, *UMOD* specifically expressed in the kidney is 470 bp, and *RBP2* specifically expressed in the duodenum is 395 bp. The original gel pictures of RT-PCR, including this figure and its repeated experiment, were provided as a source data file. **d** Examples of lncRNAs identified in this study expressed in specific tissues. H3K4me3 signals were enriched around their TSSs. **e** The distribution of H3K4me3 signals around TSSs of UCSC reference genes and newly identified transcripts. **f** Classification of enhancers based on their chromatin states (H3K27ac) among different tissues of LW pigs. **g** Statistical comparisons and representative expression profiles of genes associated with super-enhancers (top) or associated with broad H3K4me3 peaks (bottom). SE and TE indicate super- and typical enhancers, respectively. Blue shading (top) indicates SE regions; orange shading (bottom) indicates broad H3K4me3 regions (right panels). The bounds of boxplots represent the 25th percentile, median, and 75th percentile. The minima and maxima values of boxplots were defined by excluding outliners. *P*-values were calculated using a two-sided unpaired Wilcoxon test. **** indicates *P* < 2.2e−16 (*n* = 19,451 genes for super-enhancers, *n* = 178,670 genes for typical enhancers, *n* = 51,666 genes for H3K4me3 regions, and *n* = 51,666 genes for random regions). **h** Validation of the enhancers identified in this study using a Dual-Luciferase Reporter Assay System in pig 3D4/21 cells. Data shown are means ± SD (*n* = 4). * indicates *P* < 0.05, ** indicates *P* < 0.01 (3.3e−25 < *P* < 0.021), which was calculated with a two-sided Student’s *t*-test without multiple comparisons. **i** Tissue-specific enhancers from pig heart tissue carrying the conserved VISTA-validated element hs2185^43^. The numbers in brackets in ChIP-seq and RNA-seq tracks indicate signal intensity.

RNA-seq was used to analyze the transcriptomic features of 52 samples of 11 pig tissues from the four pig breeds (Supplementary Table 6 and Supplementary Fig. 3a). The RNA expression in each tissue showed distinct patterns, which were grouped into 20 clusters via the kmeans function in R (Fig. 2a and Supplementary Fig. 3b). Genes that were highly expressed in all samples were presented in the p20 cluster and were primarily involved in fundamental biological processes as revealed by the DAVID^29,30^ GO enrichment terms, indicating them to be housekeeping genes (Fig. 2a and Supplementary Fig. 3b–c). Tissue-specific expression trends were evident for more than half of these clusters (Fig. 2a and Supplementary Fig. 3b). We further identified 4510 tissue-specific genes as determined by the *Z* score matrix, where tissue-specificity was determined by at least 3-fold higher expression than other tissues in all breeds (Supplementary Data 3). Analysis of the DAVID^29,30^ GO terms showed that tissue-specific genes were significantly enriched for specific functions of various tissues (Supplementary Data 4). The typical examples and validations of these tissue-specific genes revealed a high consistency between RNA-seq and RT-PCR results, supporting the accuracy of our analysis (Fig. 2b, c, Supplementary Fig. 3d, and Supplementary Table 7).

We next identified 3316 new transcripts, including 1713 lncRNAs (based on the susScr11 assembly, see “Methods” section; Fig. 2d and Supplementary Data 5), which had not been annotated previously. We detected roughly equal numbers of new transcripts in all tissues, suggesting that early investigations into pig transcriptomes possessed limited power to detect these particular transcripts (Supplementary Fig. 3e). Furthermore, we found enriched H3K4me3 signals in the vicinities of the TSSs of these newly identified transcripts (Fig. 2d, e and Supplementary Fig. 3f), providing plausible support for their transcription. Such robust identification further confirmed the advantage of using rRNA depleted RNA and constructing strand-specific libraries, which were rarely used in early studies of pigs. Our results also showed that the tissue specificity index of our newly identified transcripts was higher than that of annotated genes using the Tau method^31,32^ in R (Supplementary Fig. 3g).

### Analysis of *cis-*regulatory elements in the pig genome

Enhancer sequences are fundamental regulators for tissue-specific gene expression and have major functional impacts on the establishment of distinct gene expression patterns^5,33^. We cataloged such tissue-specific patterns associated with putative enhancers in various pig tissues (Fig. 2f and Supplementary Fig. 4a) and identified 15,753 tissue-specific enhancers with high confidence (see “Methods” section; Supplementary Data 6). The HOMER^34^ analysis revealed that the motifs of known transcription factors with tissue-specific expressions from other species^5,35,36^ were also significantly (*P* < 10^−10^) enriched in the tissue-specific enhancers of pigs, supporting the tissue-specific functions of these enhancers (Supplementary Table 8). Results from GO analysis with the Genomic Regions Enrichment of Annotations Tool (GREAT^37^) indicated the enrichment for corresponding tissue-specific functional processes (e.g., nervous system development in the cerebrum, Supplementary Data 7), further supporting that these identified sequences do indeed function as tissue-specific *cis-*regulatory elements.

The concept of super-enhancer elements is well-established—these elements typically comprise multiple enhancer elements and represent major drivers of transcriptional activation^38,39^. In this study, 414–1306 super-enhancers were separately identified in each tissue of each breed using the ROSE algorithm^38,40^ (Supplementary Table 9 and Supplementary Fig. 4b). As expected, the expressions of super-enhancer-associated genes were significantly higher than those of typical enhancer-associated genes (Fig. 2g). Moreover, the H3K27ac intensities with super-enhancer loci also exhibited tissue-specific patterns (Supplementary Fig. 4c). Indeed, the GO enrichment analysis using GREAT^37^ indicated that the super-enhancers could largely define the identities of the respective tissue types (Supplementary Fig. 4d).

The broad H3K4me3 peaks and H3K27ac enriched active promoters were reported to induce increased gene transcriptional activation^41,42^. Results from our separate analysis revealed 418–1899 broad H3K4me3 peaks (i.e., those H3K4me3 peaks exceeding 5 kb in size) in each tissue of each breed (Supplementary Table 10). Similar to our data for the super-enhancers, the expression of genes near broad H3K4me3 peaks was significantly higher than randomly selected genes (Fig. 2g). We also separately identified 13,971–20,138 active promoters in each tissue of each breed (Supplementary Table 10). In addition, the expression of genes with active promoters was also significantly higher than the genes without H3K27ac promoters (Supplementary Fig. 5a–b). Similar to the super-enhancers, the H3K27ac intensities of active promoters also revealed tissue-specific patterns indicating their roles in driving tissue-specific gene expressions (Supplementary Fig. 5c).

To confirm the reliability and accuracy of our method, we randomly selected 15 predicted non-tissue-specific enhancers and 18 promoter sequences for validation in pig 3D4/21 cells based on the Dual-Luciferase reporter assay. The results showed significantly increased transcriptional activities by the tested enhancers and promoters compared with random genomic regions (*P* < 0.01, Student’s *t*-test; Fig. 2h and Supplementary Fig. 5d). Moreover, it was notable that 1216 of the identified enhancers harbored conserved sequences with the well-known human VISTA enhancers^43^ (Fig. 2i, Supplementary Fig. 4e, and Supplementary Data 8).

### The 3D genome structure of the pig genome

**Fig. 3:**
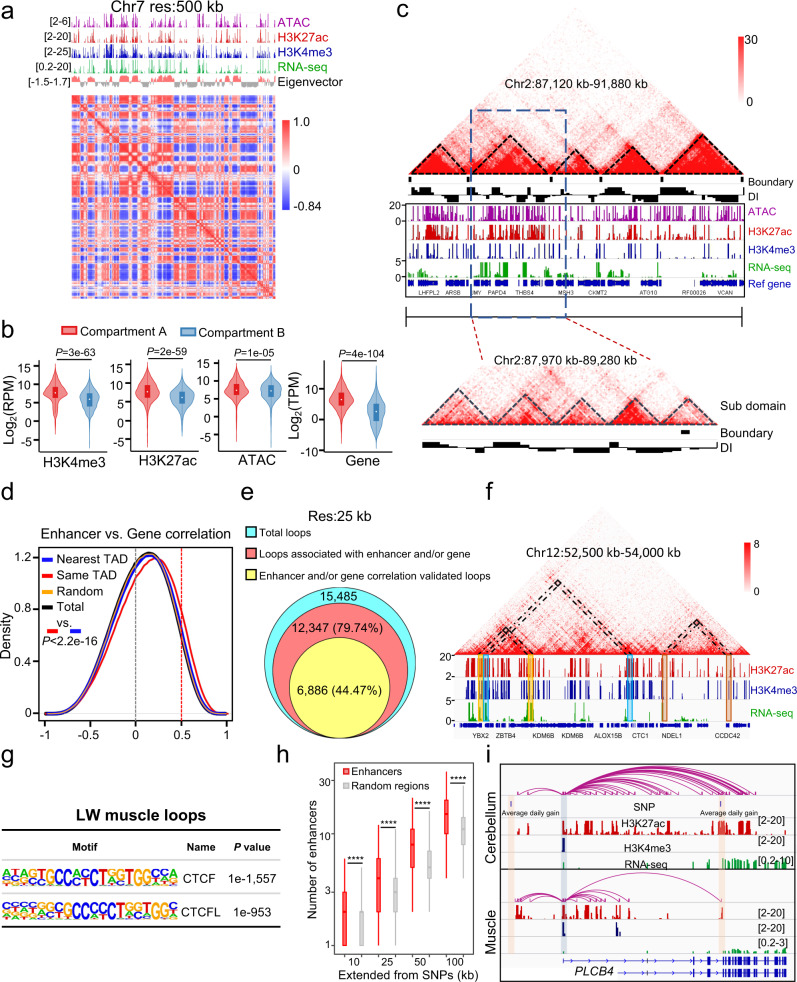
3D structure and regulation of *cis*-regulatory elements. **a** ATAC-seq, ChIP-seq, and RNA-seq enrichment and correlation map of a Hi-C matrix for chromosome 7 at 500 kb resolution (res) in LW muscle. **b** Signal intensities in the enrichment of histone modifications (H3K27ac and H3K4me3), open chromatin, and gene expression in the active “A” compartment (H3K4me3 *n* = 1167; H3K27ac *n* = 1168; ATAC-seq *n* = 1167; Gene *n* = 1004) and the inactive “B” compartment (H3K4me3 *n* = 1192; H3K27ac *n* = 1192; ATAC-seq *n* = 1191; Gene *n* = 832). A two-sided unpaired Wilcoxon test was used to calculate *P*-values. **c** TAD structure on chromosome 2 (87,120 kb–91,880 kb). Heatmap for normalized Hi-C interaction frequencies overlaid on RNA-seq data, ChIP-seq data, ATAC-seq data, directionality index (DI), and TAD boundaries. **d** Distribution of Spearman correlation coefficients between gene expression profiles and H3K27ac intensity for a given enhancer. The red vertical dotted line indicates the estimated cutoffs for significant correlation. The two-side unpaired Wilcoxon test was used to calculate *P*-value. **e** Venn diagram showing numbers of total identified Hi-C loops (blue circle), loops associated with enhancers and/or genes (red circle), and loops validated by significantly correlated enhancers and/or genes (yellow circle) at a resolution (res) of 25 kb. **f** Hi-C interaction heatmap showing consistency between the Hi-C loops and highly correlated *cis-*regulatory elements on chromosome 12 (52,500 kb–54,000 kb). The shading with the same color across tracks indicates consistency between Hi-C loops and significantly correlated enhancer-enhancer pairs, gene-gene pairs, or enhancer-gene pairs. **g** Enrichment for CTCF motifs at loop anchors. The motif enrichment was based on open chromatin regions in ATAC-seq data. The *P*-values were using a two-side cumulative hypergeometric test without adjustments. **h**, The number of enhancers detected at the indicated distances from GWAS-associated SNPs. The **** indicates *P* < 2.2e−16 and a two-sided unpaired Wilcoxon test was adopted to calculate *P*-values. The *n* = 2577, *n* = 3059, *n* = 3283, and *n* = 3378 SNPs were for extending 10 kb (*P* = 6.6e−38), 25 kb (*P* = 6.2e−98), 50 kb (*P* = 9.8e−127), and 100 kb (*P* = 1.9e−152) distances respectively. The bounds of boxplots represent the 25th percentile, median, and 75th percentile. The minima and maxima values of boxplots were defined after excluding outliners. **i** Significantly correlated enhancers and GWAS-associated SNPs around the *PLCB4* gene in pig cerebellum and muscle tissues. The orange shading indicates SNP loci and the blue shading shows *PLCB4* gene promoter regions. The purple curve lines indicate the enhancers that were significantly correlated with the *PLCB4* gene. The numbers in brackets (right side) indicate ChIP-seq, ATAC-seq, and RNA-seq signal intensities.

The Hi-C-based 3D structure of the pig genome was partitioned into either the active “A” compartments or the inactive “B” compartments following principal component analysis using the runHiCpca.pl script of HOMER^34^ (Fig. 3a). Our data illustrated that the “A” compartments were highly enriched in actively transcribed genes, active histone modifications, and open chromatin signals (Fig. 3b). Further analysis revealed distinct topologically associating domain (TAD) structures at 40 kb resolution (Fig. 3c). In total, 2364 boundaries were identified at this resolution, and collectively 2305 TADs were defined in the susScr11 genome assembly using the insulation score method^44,45^. Notably, our further comparisons revealed very little difference in the span lengths of TADs between human^46^ and pig genomes (Supplementary Fig. 6a). Moreover, sub-domains present in the TADs based on the insulation scores were also further characterized by the directionality index (DI)^47^ and TopDom analysis^48^ (Fig. 3c, bottom).

*Cis-*regulatory elements in different TADs could be insulated by boundaries^49^, and it is possible that a significant fraction of enhancers may not interact with their nearest genes, but rather with specific long-range genes^50^. Thus, we adopted the Spearman correlation coefficients (SCCs) analysis to reveal the enhancer/gene organizations of the pig genome. The results showed that SCCs of enhancer-gene pairs in the same TADs were significantly higher than those of enhancers and genes spanning the two nearest adjacent TADs (Fig. 3d), and also those of corresponding enhancer-enhancer pairs and gene-gene pairs (Supplementary Fig. 6b, c). Thus, significant enhancer-enhancer pairs (*R* > 0.5), gene-gene pairs (*R* > 0.8), and enhancer-gene pairs (*R* > 0.5) embedded in the same TAD were identified based on their SCC values (Fig. 3d and Supplementary Fig. 6b, c).

We next identified the chromatin loops via Hi-C matrix analysis. Here, 15,485 loops at 25 kb resolution and 11,838 loops at 40 kb resolution were identified by the modified HiCCUPS^51^ algorithm (Fig. 3e and Supplementary Fig. 6d). An integrative analysis of Hi-C and *cis-*regulatory element datasets revealed that, at the 25 kb resolution level, 79.74% (12,347) of the loops were associated with *cis-*regulatory elements, and 44.47% of the loops were validated by the significantly correlated *cis-*regulatory elements (Fig. 3e, f). Similar statistical results were obtained at the resolution of 40 kb (Supplementary Fig. 6d). A subsequent analysis that integrated the loop data with open chromatin regions based on the ATAC-seq data revealed significant enrichments for CTCF-binding motifs in the anchors of these loops (Fig. 3g). These results further support that the role of CTCF binding in mediating the 3D structure of mammalian genomes is strongly conserved^47,51^.

To investigate the global nature of enhancers in the regulation of complex traits in pigs, we collected published genome-wide association studies (GWASs) significantly-associated SNPs and investigated the enrichment of enhancers around them. In total, 7238 published GWAS significantly-associated SNPs were collected, and 3445 of them were non-redundant. Our results found that enhancers were significantly enriched around the GWAS significantly-associated SNPs compared with random genomic regions at different extended distances (Fig. 3h). Previous studies reported that the *PLCB4* gene represents one of the candidate genes for pig growth and average daily gain traits^52,53^. Our results extend this finding by showing the significantly-associated SNPs that were associated with an average daily gain in pigs^53,54^ were located near the significantly correlated enhancers of the *PLCB4* gene (Fig. 3i). Furthermore, the *PLCB4* gene was highly expressed in the cerebellum, and its expression was also detected in muscle tissue. These results indicated that the enhancers around the GWAS significantly-associated SNPs were potentially associated with the related pig complex traits.

### Comparison of histone changes and genome sequence variants cross different pig breeds

**Fig. 4:**
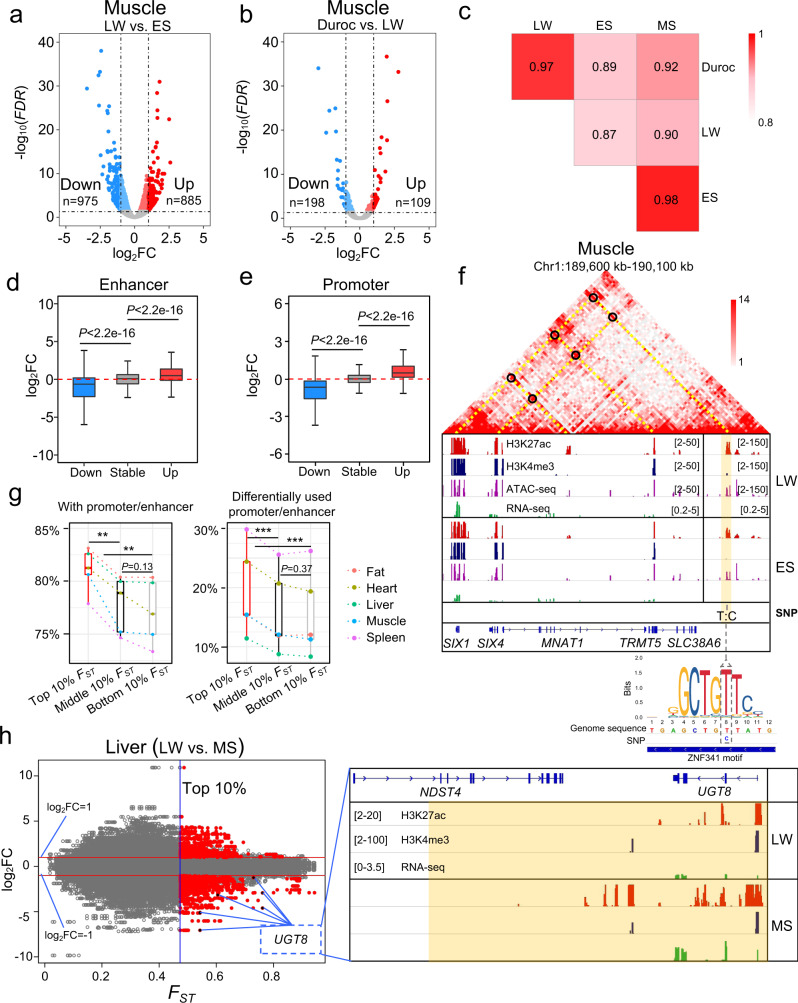
Differentially expressed genes and variable histone intensity of *cis*-regulatory elements among pig breeds. **a** The *FDR* distribution from a comparison of differential gene expression in muscle tissue of LW compared with ES pigs. **b** The *FDR* distribution from a comparison of differential gene expression in muscle tissue between LW and Duroc pigs. The red dots indicate upregulated genes, and blue dots indicate downregulated genes. **c** Pearson correlation heatmap of H3K27ac intensities at ±500 kb around all differentially expressed genes (*FDR* < 0.05) in muscle tissue of four pig breeds. **d** Boxplot of fold change (FC) in H3K27ac intensities of the enhancers (Up *n* = 2161; Down *n* = 3762; Stable *n* = 6079) of differentially expressed genes (*FDR* < 0.05 and |log_2_FC| ≥ 1) between different pig breeds in each tissue. **e** Boxplots showing fold changes in the H3K27ac intensities of active promoters (Up *n* = 2925; Down *n* = 5175; Stable *n* = 11,711) of differentially expressed genes (*FDR* < 0.05 and |log_2_FC| ≥ 1) between different pig breeds. **f** An example of a T/C SNP (Chr1:190035161) with allele frequency difference (ΔAF = 0.63) between LW and ES located in the enhancer correlated with the expression of transcription factors of *SIX1* and *SIX4*. Yellow shading indicates the region harboring the SNP, and black circles indicate Hi-C contact maps. **g** The different 10% quantile *F*_*ST*_ regions associated with *cis*-regulatory elements. ** in the left panel indicate *P* < 0.01 (*P* = 0.0042, *P* = 0.0020) and was calculated by two-sided paired *t*-test (*n* = 5). *** in the right panel indicates *P* < 0.001 (*P* = 0.00018, *P* = 0.00037) and was calculated by a two-sided paired *t*-test (*n* = 5). **h** Left: distribution of *F*_ST_ values and log_2_FC in histone modifications (H3K27ac) of *cis*-regulatory elements in liver tissue of LW and MS pigs. Right: example of histone modification signal of enhancers and promoters near the differentially expressed *UGT8* gene. Yellow shading indicates the top 10% of the *F*_ST_ genome region. *P*-values in **d** and **e** were calculated by the two-side unpaired Wilcoxon test. The bounds of boxplots represent the 25th percentile, median, and 75th percentile. The minima and maxima values of boxplots were defined after excluding outliners. The numbers in brackets located in the tracks of ChIP-seq, ATAC-seq, and RNA-seq indicate signal intensities.

It is known that major economic traits including backfat thickness and growth rate differ between Western commercial and Chinese local pig breeds. We identified 7708 non-redundant differentially expressed (DE) genes at *FDR* < 0.05, of which 4469 showed |log_2_FC| ≥ 1 (Supplementary Data 9), using the DEseq2^55^ R package and based on the gene expressions in skeletal muscle, fat, spleen, liver, and heart among the four pig breeds. In addition, more DE genes were identified through the comparison between Western commercial pig and Chinese local pig breeds than those between LW and Duroc in most tissues (Fig. 4a, b, Supplementary Fig. 7a and Supplementary Data 9).

Based on these findings, we questioned how changes in histone modifications altered differential gene expression between breeds. Taking the skeletal muscle as a representative tissue, Pearson correlation coefficients of H3K27ac signals within ±500 kb (5 kb window size) ranges of DE genes were higher between LW and Duroc than those between Western commercial (LW or Duroc) and Chinese local (ES or MS) breeds (Fig. 4c). We further investigated the fold-changes of H3K27ac intensities of enhancers, which were significantly correlated with the DE genes based on our Hi-C TADs and *cis*-regulatory elements correlation results, between different breeds using the edgeR^56^ R package. Our results showed that the changes in H3K27ac intensities of enhancers were consistent with their significantly correlated DE genes, but were different from those of the enhancers significantly correlated with randomly selected non-DE genes (Fig. 4d). Concordant results were also observed from the comparison of active promoters of DE genes (Fig. 4e). These results indicated that gene expression differences between the four breeds were associated with the differential enrichment of H3K27ac at enhancers or active promoters.

In our study, 251,361 SNPs with differential allele frequencies were localized in active promoters or enhancers based on the comparison of Western commercial pigs with Chinese local pigs. Using LW and ES pigs as a representative comparison, there was a T/C SNP (Chr1:190035161) with a difference in allele frequency (ΔAF = 0.63) located in the ATAC-seq footprint in the H3K27ac strongly-enriched enhancers (*R* > 0.66) of muscle DE transcription factors *SIX1* and *SIX4* (Fig. 4f). In addition, a G/C SNP (Chr12:5451199) with ΔAF = 0.64 was identified in the ATAC-seq footprint of the active promoter of another muscle DE gene, *ACOX1* (Supplementary Fig. 7b). Moreover, there was a possibility that the T/C SNP and the G/C SNP had a disruptive effect on the motif of muscle expressed transcription factors *ZNF341* (Fig. 4f) and *PLAGL1* in pigs (Supplementary Fig. 7b), respectively. These results suggested that these two SNPs with differential allele frequencies may be related to the activities of *cis*-regulatory elements in the DE genes *SIX1*, *SIX4*, and *ACOX1*.

We further investigated how genomic variants contributed to a histone modification and gene expression differences. We calculated the Fixation index value (*F*_ST_)^57^ between LW (*n* = 20) and MS (*n* = 16) pigs using SNPs and small indels (2–50 bp) from WGS data. The differences in genomic sequences between LW and MS were well represented in the top 10% of the changes (Supplementary Fig. 7c). When all five tissues were combined, 92.36% of the top 10% of *F*_ST_ regions overlapped with the *cis*-regulatory elements (promoters or enhancers), and 51.44% of them additionally overlapped with significantly differential *cis*-regulatory elements (H3K27ac densities, *FDR* < 0.05, and |log_2_FC| > 1) between LW and MS (Supplementary Table 11). In each tissue, 11.40–29.85% of the top 10% of *F*_ST_ regions overlapped with significantly differential *cis*-regulatory elements (Fig. 4g and Supplementary Fig. 7d). These ratios were obviously or significantly higher than that of the middle 10% of *F*_ST_ regions and the bottom 10% of *F*_ST_ regions (Fig. 4f and Supplementary Table 11).

Moreover, combining the significantly correlated enhancer-enhancer and enhancer-gene pairs in the same TADs, 18.09–50.28% of DE genes were associated with regions with differences in genome sequence and histone modification or only differences in the histone modification (Supplementary Table 12). In conclusion, our results indicated that variations in genome sequence and changes in histone modifications might both be associated with differences in gene expression between different breeds, but changes in histone modification were likely more closely related to differential expression of genes.

### The conservation of *cis-*regulatory elements among mammalian genomes

**Fig. 5:**
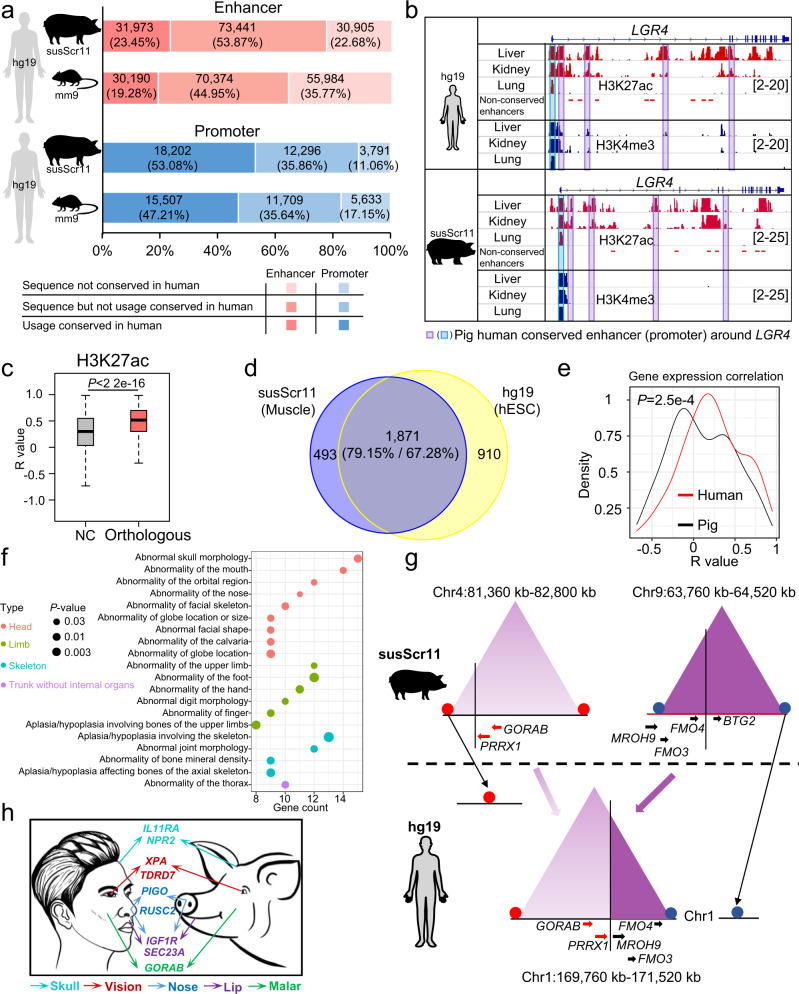
Evolutionary conservation of *cis*-regulatory elements and TADs across mammals. **a** Conservation of sequence and usage of *cis*-regulatory elements in pig and mouse genomes compared with human genomes. **b** Example of enhancers and promoters with conserved usage between pigs and humans at the *LGR4* gene locus. The numbers in brackets located in the H3K4me3 and H3K27ac ChIP-seq tracks indicate signal intensities. **c** Spearman correlations of H3K27ac intensities around pig-human orthologous gene pairs or non-orthologous genes in various pig and human concordant tissues. “NC” indicates the correlation between non-orthologous gene pairs which were random selected from all combinations of non-orthologous genes. The *P*-value was calculated using a two-sided unpaired Wilcoxon test (*n* = 14,085). The bounds of boxplots represent the 25th percentile, median, and 75th percentile. The minima and maxima values of boxplots were defined after excluding outliners. **d** The TAD boundary conservation between pig and human genomes. **e** Correlation analysis of gene expression levels in TADs that were rearranged between pigs and humans (i.e., genes in human TAD with orthologs distributed across two inter-chromosomal TADs in pig, *n* = 114 gene pairs). The Spearman correlations were calculated separately in nine corresponding tissues in pigs and humans. Correlations between orthologous gene pairs were then compared between pigs and humans using a pairwise Wilcoxon test. **f** Top 20 enriched human phenotypes of human genes in pig-human rearranged TADs. The *P*-values were using a hypergeometric test with a two-sided test and without adjustment. **g** Illustration of two pig inter-chromosomal TADs (located in susScr11 Chr4 and Chr9) rearranged into one human TAD (located in hg19 Chr1). **h** Comparison of head-related and face-related phenotypes between pigs and humans and genes within pig-human rearranged TADs associated with head-related and face-related phenotypes.

Given that a high level of sequence conservation is frequently used as evidence of conservation of functional elements, we compared the *cis-*regulatory elements between the pig, human, and mouse genomes based on human Roadmap Epigenomics^6^ and mouse ENCODE^4,5^ data using the LiftOver^58^ tool (length = 1 kb and minMatch = 0.5). We found that 77.32% of enhancers and 88.94% of promoters in the pig genome were sequence conserved in the human genome (Fig. 5a and Supplementary Table 13). Furthermore, 23.45% and 53.08% of the pig enhancers and promoters, respectively, were sequenced and usage conserved with enhancers and promoters in the human genome (Fig. 5a and Supplementary Table 13). The proportion of conserved *cis-*regulatory elements between pigs and humans was higher than that between mice and humans. Similar results were obtained when the enhancers and promoters in each tissue were compared (Fig. 5a and Supplementary Fig. 8a–d). Results from our reporter assay showed the pig enhancers and promoters, which were conserved with humans, were able to activate the reporter genes in human HEK-293T cells (Supplementary Fig. 8e–f). These results demonstrated that these sequenced conserved *cis*-regulatory elements could be activated in both pig and human cells.

Furthermore, when we compared the chromatin state annotations from Roadmap Epigenomics, we found that 37.68% of pig enhancers, which were the only sequences conserved with the human genome, were also enhancers in the corresponding human tissue at different developmental stages or in other human tissues (Supplementary Fig. 9a). In total, 61.13% of pig enhancers were confirmed to be conserved in both sequence and usage with human enhancers in various tissues. The remaining ~40% of pig candidate regulatory elements were not conserved in human genomic sequences or usages. Similar results were observed in the comparison of *cis*-regulatory elements between the mouse^4,5^ and human genomes^1^ and in the comparison of liver *cis*-regulatory elements across 20 mammalian species^25^. However, our reporter assay results showed that pig-specific enhancers and promoters exhibited activities in human HEK-293T cells and mouse C2C12 cells (Supplementary Fig. 9b–g). Thus, these results indicated that non-conserved elements could arise as species-specific events, which may play a crucial role in species characteristics.

Comparison based on a gene expression matrix showed similar expression patterns of pig-human orthologous genes (one-to-one) within each of nine pig and human concordant tissues (Supplementary Fig. 10a). Further, SCCs for gene expressions of these orthologous gene pairs were significantly higher than those between the non-orthologous genes (Supplementary Fig. 10b). We observed both conserved and non-conserved enhancers near the pig and human orthologous genes of *LGR4* and *TTR*, where similar H3K27ac histone modification patterns were observed (Fig. 5b and Supplementary Fig. 10c). Between the nine pig and human corresponding tissues, SCCs of H3K27ac intensities in the vicinity of the pig-human orthologous gene pairs (±500 kb) were significantly higher than those between the non-orthologous genes (Fig. 5c). In conclusion, we speculate that enhancers conserved in the pig and human genomes, as well as enhancers with lineage-specific sequences but similar histone (H3K27ac) modification patterns, both contributed to the similar gene expression patterns observed between pig and human concordant tissues.

### Comparison of TAD structures between the pig and human genomes

TAD boundaries can be conserved between different species^47^. The comparison of 3D genome structures between pig skeletal muscle and human embryonic stem cells (hESCs)^46^ revealed that 1871 TAD boundaries were usage conserved between pigs (79.15%) and humans (67.28%) (Fig. 5d and Supplementary Fig. 11a).

Recently, a growing body of studies has reported that the changes in 3D genome structure can help explain regulatory evolution, with especially pronounced effects on the reorganization of TADs^59,60^. By comparing the TAD structures of pigs and humans, we found that the boundaries of each of the 14 human TADs were rearranged, resulting in localization on different pig chromosomes (see “Methods” section; Supplementary Data 10). In the nine pig and human concordant tissues, SCCs of expression of genes located in the same human TADs, but on different pig chromosomes, were significantly increased in humans (Fig. 5e), a finding which indicated that the rearrangements of TADs contributed to the changes in the regulation of gene expressions between pigs and humans. Genes completely contained in the aforementioned pig-human rearranged TADs were linked to human phenotypes in the Human Phenotype Ontology (HPO) database using ToppGene^61^. Nine out of the top 20 most significant enrichments of human phenotypes (*P* < 0.05 and gene count >2) were associated with human head and face phenotypes (Fig. 5f and Supplementary Data 11). Nevertheless, we did not observe any similar HPO enriched terms from the same analysis using 14 randomly selected pig and human conserved TADs (Supplementary Fig. 11b).

Moreover, there were 19 genes linked to the human head and face phenotypes in the HPO database, which were also fully contained within eight out of the 14 TADs rearranged between pigs and humans (Fig. 5g and Supplementary Data 10). We next compared the chromosomal positions of these eight human TADs in more than 20 species to view their rearrangements. These TADs were clustered into three categories (Supplementary Fig. 11c–e and Supplementary Data 10): (i) Two TADs were rearranged between pigs and humans, containing *IGF1R* and *SEC23A* in each, bearing enriched functional annotations associated with lip and nose related phenotypes; (ii) Four TADs shared their rearrangements in 2–4 non-primate mammalian genomes and harbored a total of 11 genes, including *GORAB* and *PRRX1* with functional annotations relating to the skull, malar, and nose phenotypes; (iii) Two TADs were rearranged between primate and other mammalian genomes, and these involved six genes, including *PHOX2A*, *XPA*, and *TDRD7* that were linked primarily to the vision and face-related traits. It is worth noting that the vision of humans is better than most mammalian species^62^.

Furthermore, among the above-mentioned 19 human head and face phenotype-related genes, 12 were associated with abnormalities in the head and facial phenotypes caused by gene mutations in other species including the mouse^63^ (mutant mouse models), Zebrafish^64–66^, and Xenopus^67–69^. (Supplementary Data 10). In conclusion, these results suggested that pig-human inter-chromosome TAD rearrangements have likely contributed to the evolution of nose, mouth, and other craniofacial phenotypes (Fig. 5h).

## Discussion

In this study, we performed analyses of transcriptional activities, chromatin accessibilities, chromatin landscapes, and 3D architectures of the pig genome. Collectively, these analyses have significantly improved our understanding of the functional elements in the pig genome. Furthermore, our study adds another map of *cis-*regulatory elements for large mammals alongside the human ENCODE^1^ and Roadmap Epigenomics projects^6^. In particular, we conducted epigenetic comparisons among different pig breeds and also three mammalian species, providing critical contextualization and analysis that will facilitate improvements in both livestock (pig) breeding and human disease and biological research.

When repeat elements in an organism genome cause a small fraction of signal-artifact regions in ChIP-seq peak calling, they are termed “blacklists”^70^. However, the accurate identification of blacklists among the human, mouse, *Caenorhabditis elegans*, and *Drosophila melanogaster* genomes have been determined by more than 200 or even over 1000 input datasets^70^. In this study, only 61 input datasets of obvious small sample size were present, limiting our ability to accurately identify blacklists in the pig genome. However, it is likely that additional collection of epigenetic data will increase over time, leading to accurate identification of blacklists of the pig genome in the future.

Based on the fundamental epigenetics data from the human genome, disease-associated genetic variants were previously identified with high enrichment in specific regulatory chromatin states^71^, tissue-specific histone marks^6,72^, and open chromatin regions^73^. Through our study, we identified tissue-specific genes and enhancers in the pig genome and revealed that *cis*-regulatory elements were highly conserved between human and pig genomes. These datasets will represent a valuable resource for both the ongoing and future development of pigs as an animal model for human disease.

Previous studies based on ENCODE and Roadmap Epigenomics data have provided strong examples for the characterization of potential roles of causal mutations in *cis-*regulatory elements in gene regulation^74,75^. As such, we investigated the enhancer enrichment around pig phenotype-associated SNPs that were identified by previously published GWAS in different pig populations. Our results showed the enhancers were significantly enriched around such SNPs, indicating the potential regulatory function of these enhancers in the genetic regulation of pig complex traits.

Based on the comparison of the genomic variants between LW and MS, we discovered that over half of the genomic regions with sequence differences (top 10% *F*_ST_ regions) were embodied by their differential histone modification levels (H3K27ac). Further integration of the enhancer/gene interaction data, the regions bearing sequence variants and histone modification changes, or differing in the histone modification alone, reflect 18.09–50.28% of DE genes in corresponding tissues between LW and MS. However, our strategy bears some limitations. First, not all genomic sequence variants were embodied by the epigenetic features from these single-staged samples. Second, it is difficult to discriminate enhancer interactions with multiple target genes and other enhancers^76^, gene promoter-promoter interactions^50^, and inter-chromosome interactions^77^ when we combined the enhancer/gene interaction data and variants of the genome and histone modifications to infer the gene expression differences between LW and MS. It is likely that these limitations will be overcome with the ongoing accumulation of additional pig epigenetics data and the development of new techniques for functional genome research.

Moreover, it bears emphasizing that, compared with previous studies of genome sequence variants^21,78^, gene expression differences^22,23^ between Western commercial and Chinese local pig breeds or identification of phenotype associated SNPs using GWAS^53,54^, our study possesses enhanced utility as we combined datasets from WGS, Hi-C, histone modification, and gene expression. Collectively, this study provides a rich data resource and supports informative approaches for the interpretation of genome mutation information, such as selection sweeps and GWAS.

## Methods

### Pig tissue collection

We selected four domestic pig breeds, inclusive of two Chinese local breeds, Meishan (MS) and Enshi Black (ES), and two Western commercial breeds, Duroc and Large White (LW). The following tissue samples were collected from two male piglets at the age of two weeks per breed: skeletal muscle, spleen, heart, kidney, liver, fat, lung, pancreas, thymus, cerebrum, cerebellum, and duodenum. The samples were snap-frozen in liquid nitrogen. All experimental protocols were approved by the Ethics Committee of Huazhong Agricultural University (HZAUSW-2018-008).

### RNA-seq library construction and sequencing

Total RNA from skeletal muscle, spleen, heart, liver, and fat of two individuals from MS, ES, and Duroc pigs and total RNA from skeletal muscle, spleen, heart, kidney, liver, fat, lung, thymus, cerebrum, cerebellum, and duodenum of two LW pigs was extracted using TRIzol reagent (Thermo Scientific, 15596026). The RNA samples were of high quality with RIN values of 6.10–9.00 and 90% of them had a RIN higher than 7.00 (Supplementary Table 6). The high-quality RNA samples were further purified by the rRNA-depletion method and used to construct strand-specific RNA-seq libraries according to the protocol provided by Illumina (Illumina, San Diego, CA). The libraries were sequenced using the Illumina HiSeq X Ten (PE150) platform.

### ChIP-seq library construction and sequencing

The tissues used for ChIP-seq analysis included skeletal muscle, spleen, heart, liver, and fat from MS, ES, LW, and Duroc pigs (*n* = 2 per breed). Tissues of the kidney, lung, thymus, pancreas, cerebrum, cerebellum, and duodenum from two LW and one ES pigs were used for ChIP-seq as well. The frozen tissues were ground to a fine powder in liquid nitrogen and fixed with 1% formaldehyde (Sigma-Aldrich, 252549) at room temperature for 20 min. A final concentration of 0.2 M glycine was added to quench the fixation. Nuclear lysates were fragmented by Covaris S220 (Adaptive Focused Acoustics®). For each tissue, 3 μg of H3K4me3 (Millipore Cat# 04-745, RRID: AB_1163444) or 3 μg of H3K27ac (Abcam Cat# ab4729, RRID: AB_2118291) antibodies were mixed with 11 μL M-280 sheep anti-rabbit IgG Dynabeads (ThermoFisher, 11203D), with rotation at 4 °C for 4 h. For per immunoprecipitation, about 30 μg of chromatin was incubated with antibody–beads complexes overnight at 4 °C. ChIP-seq libraries were constructed following Illumina protocols with minor modifications (Illumina, San Diego, CA), and were sequenced on an Illumina HiSeq X Ten PE150 platform.

### ATAC-seq library construction and sequencing

Approximately 5 mg of tissue from each of the skeletal muscle, spleen, heart, liver, and fat samples was crushed into a fine powder in liquid nitrogen (Supplementary Table 1). Pulverized tissues were suspended in 1 mL ice-cold PBS and then gently spun down. The cell pellet was resuspended in 1 mL lysis buffer (50 mM HEPES with pH 7.5 [Life technologies, 15630-080], 140 mM NaCl [Ambion, AM9760G], 1 mM EDTA with pH 8.0 [Ambion, AM9260G], 10% glycerol [Sigma-Aldrich, G7757], 0.5% NP-40 [Roche, 11754599001], and 0.25% Triton X-100 [Amresco, 0694]), followed by isolation of 50,000 nuclei using a previously published protocol with minor modifications^79^. Then, the transposition reaction mix (12.5 μL TD buffer, 10 μL ddH_2_O, and 2.5 μL TDE [Illumina, FC-121-1030]) was added to the isolated nuclei. The reaction system was incubated at 37 °C for 1 h followed immediately by purification with a QIAGEN MinElute PCR Purification Kit (QIAGEN, 28006). The transposed DNA fragments were amplified with the appropriate number of cycles as determined by qPCR. Size selection of the amplified PCR products (100–600 bp fragments) was performed by gel-purification informing previously studies^79–81^. Products from the size selection step were quantified and sequenced using an Illumina HiSeq X Ten PE150 platform.

### In situ Hi-C library construction and sequencing

Skeletal muscle tissue from one LW pig was selected to do in situ HiC experiment^51^. The sample was crushed into a fine powder in liquid nitrogen and then fixed with 2% formaldehyde at room temperature for 10 min and with a final concentration of 0.2 M glycine solution to quench the reaction for 5 min at room temperature. The crosslinked tissue was mixed with 250 μL of ice-cold Hi-C lysis buffer (10 mM Tris-HCl with pH 8.0 [Invitrogen, 15567027], 10 mM NaCl, and 0.2% Igepal CA630 [Sigma-Aldrich, I8896]), and incubated on ice for 20 min. Then resuspended cell pellet in 50 μL of 0.5% SDS and incubated at 62 °C for 5–10 min. After heating, added 25 μL of 10% Triton X-100 (Sigma, 93443) with 145 μL of water to quench the SDS and incubated at 37 °C for 15 min. Chromatin was digested with 100 U of *Mbo*I restriction enzyme (NEB, R0147) overnight at 37 °C followed by labeling of the DNA fragment ends with biotin (37.5 μL of 0.4 mM biotin-14-dATP [Life Technologies, 19524-016], 1.5 μL of 10 mM dCTP, 1.5 μL of 10 mM dGTP, 1.5 μL of 10 mM dTTP, 8 μL of 5U/μL DNA Polymerase I [NEB, M0210]) through incubation at 37 °C for 1.5 h. DNA ligation was performed with T4 DNA ligase (NEB, M0202) by incubating the reaction at room temperature for 4 h with slow rotation. The biotinylated DNA was sheared to a size of 300–500 bp with Covaris S220. Libraries were constructed following Illumina protocols (Illumina, San Diego, CA) and sequenced on an Illumina HiSeq X Ten PE150 platform.

### Dual-Luciferase reporter assay

The predicted promoters and enhancers were verified by a Dual-Luciferase Reporter Assay System^82^. The chromosome coordinates, primer sequences, and linker sequences are shown in Supplementary Data 12. The cloning was performed with the pGL3-Promoter plasmid (Promega, E1761) which was digested with BamHI and SalI (NEB), and inserted a linker sequence. Besides, pGL3-Basic plasmid (Promega, E1751) was digested with MluI and BglII (New England Biolabs) and inserted a linker sequence (Supplementary Data 12). The 13 pig-specific enhancers (sequence not conserved with human and with the mouse), 15 pig-human conserved enhancers (both sequence and usage conserved with humans), and 14 randomly selected negative genomic regions were assayed. The pig genomic DNA was used as a template to amplify these enhancers and random genomic regions with a length of around 2 kb each. The amplified products were separated on a 1% agarose gel, purified, and then cloned into the modified pGL3-Promoter plasmid. In addition, 11 pig-specific promoters (sequence not conserved with human and with the mouse), 18 pig conserved promoters (both sequence and usage conserved with humans), and 14 randomly selected negative genomic regions were validated. Next, 2 kb sequences around the center of the above-selected regions were amplified using pig genomic DNA as the template. The amplified products were used to construct into the modified pGL3-Basic plasmid.

Pig 3D4/21 macrophage cells (ATCC Cat# CRL-2843, RRID: CVCL_0F14), human HEK-293T cells (ATCC Cat# CRL-3216, RRID: CVCL_0063), and mouse C2C12 cells (ATCC Cat# CRL-3419, RRID: CVCL_UR38) were transfected using Lipofectamine 3000 (Invitrogen, L3000015) and Opti-MEM (Gibco, 11058021) and incubated for 24 h with four technical replicates for each construct. Fluorescence values were determined using a Dual-Luciferase® Reporter Assay System (Promega, E1910), according to the manual.

### Reverse transcription PCR (RT-PCR)

Total RNA from the spleen, skeletal muscle, liver, heart, fat, thymus, lung, kidney, duodenum, cerebellum, and cerebrum of LW pigs was extracted using TRIzol reagent (Thermo Scientific, 15596026). Then 1ug RNA was performed to do reverse-transcription with RevertAid First Strand cDNA synthesis kit (K1621, Thermo Scientific, USA) following the instruction. Next, four folds diluted cDNA was used for PCR amplification, and primer information was listed in Supplementary Table 7.

### Sequencing data analysis

To process the basic sequencing data from RNA-seq, ChIP-seq, and ATAC-seq, we followed the online guidelines provided by the ENCODE project (https://www.encodeproject.org/) with minor modifications.

### RNA-seq

#### Mapping and assembly

The reads were mapped to susScr11 (*Sus scrofa* 11.1) reference genome assemblies using TopHat v2.1.1^83^. The transcript assembly of each sample was performed separately by using a BAM file as the input file for Cufflinks v2.2.1^84^. The resulting individual transcript assemblies were merged further to a single transcript assembly using the Cuffcompare utility, which was included with the Cufflinks package v2.2.1^84^.

#### Identification of lncRNAs and other types of transcripts

To identify lncRNAs, the transcripts from the single transcript assembly were filtered by the following steps: (i) the identified transcripts, which must have class code “x”, “i”, or “.” in at least two samples, but did not overlap with any known transcripts or with class code “u” transcripts; (ii) transcripts with length ≥200 bp and exon number ≥2; (iii) transcripts with FPKM ≥1 and reads ≥5 in at least two replicates; (iv) the identified transcripts without potential coding regions, which were filtered using CNCI v2.0^85^ and CPC v2.0^86^.

Next, we identified other types of new transcripts of the pig genome. At first, we selected the transcripts that were assembled in two replicates in the transcript assembly file of each sample. Second, the filtered transcript assembly files were merged using the Cuffmerge program of Cufflinks v2.2.1^84^. Then, the Cuffcompare utility of Cufflinks v2.2.1^84^ was applied again to integrate the merged transcript assembly file and the lncRNA GTF file. The new GTF file was used to calculate transcript expression levels using RSEM v1.3.0^87^. The last step was to filter the transcripts to identify new transcripts by the following criteria: (i) transcripts on the same strand of known transcripts and exons overlapped with any known transcripts were omitted; (ii) lncRNAs which were identified before were removed; (iii) transcripts with class code “x”, “i”, or “u” and with FPKM ≥5 at least in any two replicates.

We further calculated the H3K4me3 intensities near to lncRNAs and other types of newly identified transcripts via the computeMatrix function of deepTools v2.0^88^, and created a profile plot by using the plotProfile function of deepTools v2.0^88^. The ChIP-seq data were processed using the procedures described below in the “ChIP-seq” section. Then, the newly identified transcripts with protein-coding capacity were selected by CPC v2.0^86^ and compared with the NCBI Reference protein database of humans (taxid:9606) using BLASTX v.2.6.0^89^. The cutoff of BLAST results was set as EXPECT threshold <10^−5^.

#### Calculating gene expression and identifying tissue-specific genes

The GTF file of lncRNAs and other types of newly identified transcripts was integrated with the annotation file from UCSC to calculate gene expressions using RSEM v1.3.0^87^. Genes with a mapped read count >4 in at least two samples were used for further analysis. The TPM profile was processed with normalization via the qsmooth package v1.4.0^90^ in R v3.6.3. A heatmap that showed distinct patterns of expression in each tissue was clustered and plotted using the kmeans function and pheatmap package v1.0.12^91^ in R v3.4.3, respectively.

To identify tissue-specific genes, the genes with low expression (TPM < 1) in all samples were further excluded. Then, the TPM matrix of gene expression was normalized using qsmooth package v1.4.0^90^. After the calculation of average expression between replicates, the normalized expression matrix was transformed into a *Z* score matrix using the Scale function in R v3.4.3. According to the *Z* score matrix, genes with at least three times higher expression across each sample of a given tissue type from different breeds than the expression across all samples of each other tissue type from different breeds were classified as tissue-specific genes. Considering the negative values of the *Z* score matrix, we adopted the following comparison method. When the two values of comparison contained positive and negative values, the fold would be default counted as 3 or −3. When the two values of comparison are all negative values, the fold would be counted as the reciprocal of the result. We conducted GO enrichment analysis on tissue-specific genes using DAVID^29,30^, and the significance level was set as DAVID modified Fisher Exact *P-*value <0.01. The tissue specificity index of known genes (from UCSC Ensembl gene) and newly identified transcripts, which was plotted in Supplementary Fig. 3g, was calculated according to a previous Tau method^31,32^ with a custom R script in R v3.4.3.

### ChIP-seq

#### Mapping

ChIP-seq datasets were processed with reference to the ENCODE ChIP-seq pipeline (https://github.com/kundajelab/chipseq_pipeline). The ChIP reads and Input reads were aligned to susScr11 and susScr3 genome assemblies using BWA v0.7.15^92^. The filtered BAM files were generated by removing low MAPQ reads (<25), unmapped reads, mate unmapped reads, not primary alignments, reads failing platform, and duplicates using SAMTools v1.9^93^ and Picard v1.126 (https://broadinstitute.github.io/picard). Also, sequenced reads in the filtered BAM files were defined as effective reads for ChIP-seq datasets. Narrow peaks and bdg signal tracks were generated with MACS2 v2.1.0^94^ (-g genome.size -p 0.01 --nomodel --shift 0 --extsize n --keep-dup 1 -B --SPMR). The “n” for the extend size parameter was calculated automatically by the ENCODE ChIP-seq pipeline. The “genome.size” was the sum calculated from the chromosome size files downloaded from the UCSC browser, which were named as “susScr11.chrom.sizes” or “susScr3.chrom.sizes”.

#### Quality control

The cross-correlation values, including normalized strand coefficients (NSC) and relative strand correlations (RSC), were calculated for each sample via the ENCODE ChIP-seq pipeline as well. The fraction of reads in peaks (FRiP) scores was calculated based on read counts (FRiP = reads falling into peak regions/total mapped reads). Samples with NSC > 1.05, RSC > 0.8, and FRiP > 1% were considered to be qualified^95^.

For saturation analysis, we ranked all 61 H3K27ac ChIP-seq samples by their numbers of effective reads. Then, one sample of each condition was chosen randomly from the 25% quantile, the 25–75% quantile, and the 75% quantile, respectively, to evaluate the saturation of sequencing depth. The narrow peaks for a series of subsamples (10%, 20%, 30%, …, 80%, 90%) down-sampled from the effective reads for each ChIP sample were called using the aforementioned ENCODE pipeline. The same Input subsample, which was down-sampled (equal to the minimum size of chosen samples) from the effective reads for each Input sample, was used as a control for total subsamples of the same ChIP sample. The saturation plot was generated based on the significant peak number (*P* < 10^–5^) and the fraction of effective reads. Moreover, the saturation analysis for H3K4me3 ChIP-seq samples was processed by the same method as in the above description. TSS enrichment scores for each sample were calculated and plotted by using the ENCODE script (https://github.com/ENCODE-DCC/chip-seq-pipeline2/blob/master/src/encode_task_tss_enrich.py). The cutoff value of the TSS enrichment score (>5) referred to the ATAC-seq standard of ENCODE (https://www.encodeproject.org/atac-seq).

#### Data reproducibility and peak calling

Read coverage of genomic regions for filtered BAM files was computed to assess the genome-wide similarities of replicate BAM files with 2 kb bin size by multiBamSummary bins function in deepTools v2.0^88^. The read coverage matrix was depth normalized (RPM, reads per million mapped reads) followed by quantile normalization with the normalize.quantiles function of preprocessCore package v1.40.0^96^ in R v3.4.3. The resultant matrix was then used to perform principal components analysis with prcomp function in R v3.4.3. In addition, the Pearson correlation coefficients between any two replicates were estimated based on the read coverage matrix resulting from the above multiBamsummary step. If the Pearson correlation coefficients of read coverage were >0.83, the replicate BAM files were merged as one ChIP BAM file or Input BAM file for peak calling with the aforementioned ENCODE pipeline as well. Peaks were further processed by following four steps: (i) narrow peaks with *P* > 10^–5^ were filtered out; (ii) read coverage of 2 kb region centered at the midpoint of peaks was calculated with multiBamSummary BED-file function in deepTools v2.0^88^ and normalized with read depth (IP_RPM_ = IP_peak region total reads_/IP_total mapped reads per million_; INPUT_RPM_ = INPUT_peak region total reads_/INPUT _total mapped reads per million_); (iii) the enriched regions for H3K4me3 and H3K27ac were defined with more than a two-fold change of normalized read coverage (IP_RPM_ > 2 × INPUT_RPM_) and with normalized read coverage change (IP_RPM_-INPUT_RPM_) > 1; and (iv) the overlap of enriched regions were merged, and then the 2 kb regions centered at the midpoint of merged regions were acquired to identify enhancers and promoters.

#### Identification of *cis*-regulatory elements

The *cis-*regulatory elements, including enhancers and promoters, were identified in each tissue based on the above-enriched regions. The enriched regions for H3K4me3 were defined as potential promoters. The enriched regions for H3K27ac overlapped with neither the extended regions (2.5 kb upstream and 1 kb downstream) of a gene transcription start site (TSS), which was downloaded from UCSC Table Browser and had miRNA removed nor the enriched regions of H3K4me3 were finally designated as enhancers. Super-enhancers of each breed and tissue were identified in accordance with ROSE default parameters from the Young Lab^38,40^.

Moreover, the enriched regions of H3K27ac, which overlapped with a flanked TSS region (upstream 2.5 kb and downstream 1 kb) or a potential promoter, were identified as active promoters. The broad H3K4me3 peaks were identified by referring to Chen’s method^41^ based on H3K4me3 broad-peaks detected with MACS2 v2.1.0^94^ (-g genome.size -p 0.01 --nomodel --shift 0 --extsize n --keep-dup 1 --broad) and with minor threshold modification including: (i) the cutoff for *P* < 10^–8^, fold change >4, and peak width >5 kb were adopted to filter peaks; and (ii) the filtered peaks that overlapped with TSS extended regions, including UCSC-annotated genes and newly identified transcripts in our RNA-seq analysis, were identified as the broad H3K4me3 peaks. For each tissue and breed, identification of all *cis*-regulatory elements was performed independently.

#### Comparison with previous studies

The H3K27ac/H3K4me3 ChIP-seq data of pig liver and induced pluripotent stem cells were downloaded from the NCBI dataset (Supplementary Table 5) and processed using the same methods as described above to identify enhancers and promoters. Promoters identified from these data and extended regions of the TSS site as described above were used as the known promoters. The intersect command of BEDTools v2.26.0^97^ was used to detect the overlap of *cis*-regulatory elements from the previous and present research with the parameter “-f 0.1 -r”.

#### Identification of tissue-specific enhancers

The 2 kb enhancers from different tissues of each breed were merged and extended around their center into a 2 kb region. These whole regions were defined as total enhancers. For the total enhancer regions, ChIP (H3K27ac) reads counts and Input reads counts were counted with multiBamSummary BED-file function in deepTools v2.0^88^, and normalized read coverage changes (IP_RPM_–INPUT_RPM_) were calculated as described above. Then, the matrix of normalized read coverage changes for total enhancers in each tissue from each breed was normalized with the normalize.quantiles function of preprocessCore package v1.40.0^96^ in R v3.4.3. The enhancers with greater than 1 quantile-normalized read coverage change in the same tissue from different breeds but less than 0.5 in any other tissues were defined as tissue-specific enhancers.

#### Gene ontology enrichment analysis of tissue-specific enhancers

The *cis*-regulatory element positions were converted to those corresponding to the human genome (hg19) using LiftOver^58^ utility from UCSC with “minMatch = 0.1” and other default parameters. The enrichment analysis of GO Biological Process for these converted *cis*-regulatory element regions was performed using the Genomic Regions Enrichment of Annotations Tool (GREAT v 3.0.0^37^).

#### Motif analysis in the tissue-specific enhancer regions

The motif analysis of enhancers was performed using the following steps: (i) the total H3K27ac narrow peaks of each tissue for total enhancers were merged using BEDTools v2.26.0^97^ and used to find motifs with HOMER’s findMotifsGenome.pl v4.10.3;^34^ (ii) the enhancer matrix of normalized read coverage change (without quantile-normalized), which was calculated as described in the “Identification of tissue-specific enhancers” section, was sorted according to the average value of the same tissue across all pigs; (iii) for each tissue, the corresponding H3K27ac ChIP narrow peaks of the above top 5000 enhancers were further sorted based on *P*-value and then the top 3000 narrow peaks were used to find motifs with HOMER’s findMotifsGenome.pl v4.10.3^34^, separately; and (iv) the total enhancer motifs with *P* < 10^−10^ in at least one tissue and their corresponding transcription expressed in corresponding tissue (TPM > 1) were considered significantly enriched motifs, and similar motifs were merged by TOMTOM v4.11.2^98^ with *P* < 10^−5^.

#### Analysis of H3K27ac intensity patterns at super-enhancer regions across different tissues of four pig breeds

The analysis of H3K27ac modification patterns of super-enhancers across different tissues was performed using the following steps: (i) the super-enhancers from different tissues of the four pig breeds were merged as total super-enhancers; (ii) the read coverage of H3K27ac per total super-enhancer in each tissue of each pig breed was calculated using the multiBamSummary BED-file function of deepTools v2.0^88^; (iii) the normalized matrix of H3K27ac intensity was determined using IP_RPKM_-INPUT_RPKM_, followed by normalization with the normalize.quantiles function of preprocessCore package v1.40.0^96^ in R v3.4.3; and (iv) clustering and plotting of H3K27ac modification patterns of super-enhancers were performed using the kmeans function and the pheatmap package v1.0.12^91^ in R v3.4.3, respectively.

#### Analysis of H3K27ac intensity patterns at active promoters

Genes that were detected by RNA-seq and with TSS regions or potential promoter regions that overlapped with H3K27ac enrichment regions were defined as active promoter-associated genes. The remaining genes detected by RNA-seq were defined as without H3K27ac promoter-associated genes. The analysis of H3K27ac modification patterns of active promoters in each tissue of LW pigs was performed using the following steps: (i) the active promoters from each tissue of LW pigs were merged and extended around their center into 2 kb regions which were defined as total active promoters; (ii) the read coverage of H3K27ac per total active promoter was calculated using the multiBamSummary BED-file function in deepTools v2.0^88^; (iii) the normalized matrix of H3K27ac intensity was determined using IP_RPM_-INPUT_RPM_, followed by normalization using the normalize.quantiles function in preprocessCore packages v1.40.0^96^ in R v3.4.3; and (iv) clustering and plotting of H3K27ac normalized intensity patterns of active promoters were performed using the kmeans function and the pheatmap package v1.0.12^91^ in R v3.4.3, respectively. Heatmaps of enhancer matrices were plotted with the same method.

### ATAC-seq

#### Mapping and peak calling

ATAC-seq datasets were processed with reference to the ENCODE ATAC-seq pipeline (https://github.com/kundajelab/atac_dnase_pipelines). After checking and trimming the adapter with Cutadapt v1.14^99^, the ATAC-seq reads were mapped to pig susScr11 and susScr3 reference genome assemblies using Bowtie2 v2.3.4.1^100^. Low MAPQ reads (<25), unmapped reads, mate unmapped reads, not primary alignments, reads failing platform, and duplicates of mapped BAM files were removed using SAMTools v1.9^93^ and Picard v1.126 (https://broadinstitute.github.io/picard). The effective reads were generated by further removing mitochondrial reads using BEDTools v2.26.0^97^ to call peaks. The peak for each replicate was called individually with MACS2 v2.1.0^94^ (-g genome.size -p 0.01 --nomodel --shift -75 --extsize 150 -B --SPMR --keep-dup all --call-summits) following the guidelines of the ATAC-seq pipeline from the ENCODE project (https://github.com/kundajelab/atac_dnase_pipelines). The same “genome.size” was used as in ChIP-seq processing.

#### Quality control

The cross-correlation values were calculated for each sample using the above ATAC-seq pipeline (https://github.com/kundajelab/atac_dnase_pipelines). The thresholds of NSC and RSC referred to ChIP-seq. The FRiP scores for each sample were calculated with the procedure mentioned pertaining to ChIP-seq. Samples with FRiP > 0.2 were considered to be qualified (https://www.encodeproject.org/atac-seq/).

For saturation analysis, we ranked all 25 ATAC-seq samples by their numbers of effective reads. Next, we randomly chose one sample from the 25% quantile, the 25–75% quantile, and the 75% quantile, respectively, to evaluate the saturation of sequencing depth. The narrow peaks for a series of subsamples (10%, 20%, 30%, …, 80%, 90%) of the effective reads for each sample were called using the aforementioned ATAC-seq pipeline. The significant peak number (*P* < 10^–5^) was plotted based on the fraction of effective reads. TSS enrichment scores for each sample were calculated and plotted by using the ENCODE script (https://github.com/ENCODE-DCC/atac-seq-pipeline/blob/master/src/encode_task_tss_enrich.py). The cutoff value of the TSS enrichment score (>5) for high-quality data referenced ENCODE (https://www.encodeproject.org/atac-seq).

#### Analysis of data reproducibility and open chromatin region

Pearson correlation coefficients between replicates were calculated based on read coverages of genomic regions, which were computed with 2 kb bin size using the multiBamSummary bins function in deepTools v2.0^88^ with filtered BAM files of each replicate. Principal components analysis was performed as described in the “Data reproducibility and peak calling” section. The significant narrow peaks (*P* < 10^−5^) of each sample were used for further analysis. Then, the filtered peaks of replicates with high Pearson correlation coefficients (*R* > 0.85) were merged with BEDTools v2.26.0^97^ (at least 50% overlap with each peak between two replicates). The total peak positions after merging were defined as open chromatin regions. The highly correlated replicate BAM files (*R* > 0.85) were merged to generate signal tracks with MACS2 v2.1.0^94^ as well.

#### Footprint analysis

The footprint analysis was primarily performed as the following steps: (i) the broad peak of merged data was called using the MACS2 v2.1.0^94^ broad module, and a significant broad peak was defined as *P* < 10^-5^; (ii) the footprint of each significant broad peak was analyzed using TOBIAS^101^ v0.10.1 and vertebrates motif from JASPAR2020 with parameter “--motif-pvalue 5e-4”; and (iii) the significant bound motif were filtered with scores >5 and TPM > 1 in corresponding tissue.

### Hi-C

#### Mapping and matrix generation

First, the configuration file for Hi-C processing was prepared with corresponding BOWTIE2_IDX_PATH, GENOME_SIZE, and GENOME_FRAGMENT (BIN_SIZE = 40000; LIGATION_SITE = GATCGATC). The paired-end Hi-C reads from different libraries were mapped separately to the pig genome (susScr11) using HiC-Pro^26^ pipeline v2.9.0 (https://github.com/nservant/HiC-Pro) with the parameter “-s mapping”. After mapping, the singleton, multi mapped, dumped, dangling, and self-circle paired-end reads and PCR duplication were all removed using HiC-Pro^26^ pipeline v2.9.0 with the parameter “-s proc_hic”. Next, files with.bwt2pairs and.validPairs suffixes from different libraries were moved to a new folder to become the input data for subsequent analysis. Then, we merged multiple libraries, built raw inter-/intra-chromosomal contact maps, and ran ICE normalization on contact maps using HiC-Pro^26^ pipeline v2.9.0 in order of parameters “-s merge_persample”, “-s build_contact_maps”, and “-s ice_norm”. The visualized contact matrices were generated at resolutions of 1 kb, 5 kb, 10 kb, 25 kb, 40 kb, 50 kb, 100 kb, and 1 Mb by using juicer tools v1.8.9^102^ (https://github.com/aidenlab/juicer/wiki/Download).

#### A/B compartment and normalized interaction matrix

The BAM files of read1 and read2 from different libraries were merged as single one read1 BAM and read2 BAM using SAMTools v1.9^93^, respectively. The makeTagDirectory command of HOMER^34^ with default parameters was used to process the alignment file into the HOMER-style tag directory. Principal Component Analysis (PCA) was performed to further reveal active (“A” compartment) and inactive (“B” compartment) chromatin regions along the genome with the runHiCpca.pl script of HOMER^34^ following the parameters “-res 40000 -genome susScr11”. The output file with.PC1.bedgraph suffix was loaded directly to the Integrative Genomics Viewer (IGV v2.4.18^103^) to view the partition of chromatin compartments. For visualization of the interaction matrix, the HOMER^34^ analyzeHiC command was used to create a normalized interaction matrix from the tag directories with the parameters “-chr -res 500000 -corr”, consequently, each chromosome corresponded to a matrix that was used to visualize as a heatmap with Treeview3 (https://bitbucket.org/TreeView3Dev/treeview3/).

#### Insulation score calculation and TAD calling

The TAD structure (insulation/boundaries) was defined by the insulation method that was developed by previous studies^44,45^. The matrix that was used to calculate the insulation score was normalized via the ICE method^104^ for discarding biases from the raw matrix. The insulation score of the ICE matrix was calculated using the following options: -is 480000 -ids 320000 -im iqrMean -ss 160000. The subdomain calling adopted TopDom v0.0.2^48^ following “window.size=3”.

#### DI calculation

The directionality index (DI) of the 40 kb raw Hi-C matrix was calculated according to a previous study^47^.

#### Hi-C loop calling

Loops of Hi-C data were analyzed by HiCCUPS^51^ with minor modifications. The resolution parameter was set as “-r 25000, 40000”.

#### Correlation of *cis*-regulatory elements

The gene expression matrix containing the average TPM values of two replicates and the matrix of H3K27ac normalized read coverage changes (IP_RPM_-INPUT_RPM_, calculated from the “Identification of tissue-specific enhancers” section) of enhancers were arranged by the same information of tissue and breed. Then, the intra-chromosome SCCs of enhancer-enhancer pairs, enhancer-gene pairs, and gene-gene pairs were calculated according to these two matrices using Pearson3 (https://github.com/cphill25/Pearson) with parameters “-s -w 0”. The density curve plot for SCCs of pairs was produced with the density function in R v3.4.3, including the pairs within the same TAD or nearest TAD and the random pairs with the equal number of the same TAD.

### Identification of short variants

In total, we downloaded 491 pig whole genome sequences from public databases which were also collected in our previous study^105^. The raw sequencing data were converted to fastq files by using SRAToolkit v2.8.2^106^, and the fastq files were trimmed by removing adapters and low-quality bases using Trimmomatic v0.36^107^. Next, the remaining high-quality reads were aligned to the Sscrofa11 reference genome assembly using BWA v0.7.17^92^. Finally, the high-quality aligned reads with few mismatches (<6) were used for short variant detection. The highly confident short variants were obtained by employing GATK v4.0.3.0 (https://software.broadinstitute.org/gatk/) variant calling pipelines, according to the GATK best practice online documentation. The data were further filtered by GATK based on the following criteria: (i) “-selectType INDEL --minIndelSize 2 --maxIndelSize 50 -selectType SNP”; (ii) “QD < 20.0 | | DP < 5 | | MQ < 10.0 | | QUAL < 20.0 | | ReadPosRankSum < −8.0 | | FS > 10.0”. Finally, both the SNP and small Indel VCF files were then further filtered using VCFtools v0.1.15^108^ with “-maf 0.0407” (20 individuals).

### Identification of SNPs with differential allele frequencies between different breeds

Allele frequency differences (ΔAF) were calculated from the comparison of Western commercial pigs (*n* = 195) with Chinese local pigs (*n* = 177) and with a comparison of LW and Duroc pigs (*n* = 79) with MS and ES pigs (*n* = 18) using VCFtools v0.1.15^108^. SNPs with high ΔAF (>0.6) between Western commercial pigs and Chinese local pigs, and between LW and Duroc pigs and MS and ES pigs, were determined as SNPs with differential allele frequencies.

### Comparative analysis between different pig breeds

#### Analysis of differentially expressed genes and differential *cis*-regulatory elements among the four breeds

DE genes between breeds were identified separately for each tissue (skeletal muscle, spleen, heart, liver, and fat) and the reads count of the gene expression matrix were used as input in DEseq2^55^ in R v3.4.3. The H3K27ac intensity around (±500 kb) DE genes (*FDR* < 0.05), between any two breeds of pig muscle, was calculated using changes in muscle normalized H3K27ac read coverage relative to Input (IP_RPM_–INPUT_RPM_) with 5 kb window size by multiBamsummary BED-file function of deepTools v2. 0^88^. We next calculated the Pearson correlation coefficients between four pig breeds using the above-calculated H3K27ac intensity with cor function in R v3.4.3.

The differential enhancers between any two breeds for each tissue (skeletal muscle, spleen, heart, liver, and fat) were identified by the following steps: (i) the enhancers with changes in normalized H3K27ac read coverage (IP_RPM_-INPUT_RPM_, calculated from “Identification of tissue-specific enhancers” section) less than 1 in two breeds were removed; (ii) the enhancers which overlapped with super-enhancers in corresponding breeds were removed; (iii) the remaining enhancers underwent differential analysis using package edgeR^56^ in R v3.4.3 with H3K27ac reads counts of two replicates from any two breeds as input data, which was computed via the multiBamsummary BED-file function of deepTools^88^ v2.0; and (iv) the significant cutoff was set at *FDR* < 0.05 and |log_2_FC| > 1. Moreover, the differential active promoters between any two breeds for each tissue (skeletal muscle, spleen, heart, liver, and fat) were identified with a similar method except for the super-enhancer filtering step.

Based on DEseq2 results, we classified genes into three categories, which included upregulated genes (log_2_FoldChange ≥ 1 and *FDR* < 0.05), downregulated genes (log_2_FoldChange ≤ −1 and *FDR* < 0.05), and stable genes. Enhancers associated with genes were filtered based on our significantly correlated enhancer-gene pairs within the same TAD. The accumulated log_2_FoldChange values of enhancers and active promoters from the edgeR results above, which were associated with upregulated genes, downregulated genes, or random selected stable genes, were used for plotting boxplots.

#### *F*_ST_ value calculation

The *F*_ST_ value between LW (*n* = 20) and MS (*n* = 16) was calculated by VCFtools v0.1.15^108^ with parameters “--fst-window-size 150000 --fst-window-step 15000”.

### Human Roadmap Epigenomics and mouse ENCODE data

Human ChIP-seq data for H3K27ac, H3K4me1, and H3K4me3 of 11 tissues (heart, liver, spleen, kidney, lung, thymus, intestine, brain, fat, skeletal muscle, and pancreas) were downloaded from the Roadmap Epigenomics project^6^. The processing of ChIP-seq data and identification of *cis-*regulatory elements on the hg19 genome were consistent with the methods described above for pigs, except that the identification of enhancers was based on signaling of either H3K27ac or H3K4me1, in agreement with the identification of enhancers in the mouse ENCODE project. Mouse promoters and enhancers on the mm9 genome from nine tissues (heart, liver, spleen, kidney, lung, thymus, intestine, cerebrum, and cerebellum) were downloaded from the YUE lab. Total enhancers or promoters from different tissues were merged to produce 2 kb regions of midpoint extension within the same species.

### Conservation analysis of *cis*-regulatory elements across mammals

The centered 1 kb of each predicted promoter and enhancer were converted from pig/mouse genomic locations (susScr11/mm9) to human genomic locations (hg19) by using the LiftOver^58^ tool from the UCSC genome browser with the parameter “minMatch = 0.5”. The enhancers (promoters) that could be converted to human genomic locations were considered as sequence conserved enhancers (promoters). Furthermore, the enhancers (promoters) were considered usage conserved if the corresponding human homologous sequence was covered by human enhancers (promoters).

The pig enhancers which were conserved in sequences but not in usage with human enhancers were further overlapped with human chromatin states downloaded from the Roadmap Epigenomics project (https://egg2.wustl.edu/roadmap/data/byFileType/chromhmmSegmentations/ChmmModels/imputed12marks/jointModel/final/).

Total enhancers from 12 pig tissues that converted to the hg19 genome were compared with enhancers from the VISTA Enhancer Browser^43^. The overlapped enhancers were considered experimentally validated human enhancers.

### The TAD conservation analysis between pig and human genomes

The pig TAD boundaries were converted from pig genomic locations (susScr11) to human genomic locations (hg19) by using the LiftOver^58^ tool with the parameter “minMatch = 0.1”. Then, the converted pig boundary locations on hg19, which overlapped with extended human TAD boundaries (±100 kb) using the BEDTools v2.26.0^97^ intersect command with the parameters “-f 0.1 -r”, were defined as pig-human conserved boundaries. On the bias of our statistics results in Supplementary Fig. 11a, the converted pig boundary locations on hg19 were further defined as pig-human conserved boundaries because they were located within 500 kb of their nearest human boundaries and without other conserved pig-human boundaries. The human TADs with the following criteria were defined as pig-human rearranged TADs: the boundaries on each side of a human TAD were conserved with pig boundaries from two different chromosomes.

The comparison of chromosome locations at the pig-human rearranged TAD regions was performed using the data from UCSC multiple alignments of the following assemblies to the human genome, which was downloaded from http://hgdownload.cse.ucsc.edu/goldenPath/hg19/multiz100way/.

### The conservation analysis of pig and human orthologous genes

The orthologous genes between pigs and humans were downloaded from Ensembl BioMart, and one-to-one pig-human orthologous genes were used for further analysis. The TPM expression matrix of human tissues of these orthologous genes was downloaded from GTEx Database^109^. Then, we plotted the heatmaps of the expression patterns of these orthologous genes in each tissue of pigs and humans using the previous pig cluster results from the K-means cluster analysis.

The expression profiles of these orthologous genes in various pig and human concordant tissues were processed separately by qsmooth package v1.4.0^90^ in R v 3.6.3. The positions of orthologous genes were expanded to ±500 kb, and H3K27ac intensity of each pig and human concordant tissue around the expanded regions was calculated by multiBamsummary BED-file function in deepTools v2.0^88^. The expression and H3K27ac intensity profiles of orthologous genes were used to calculate SCCs of one-to-one orthologous and non-orthologous gene pairs using program pearson3 (https://github.com/cphill25/Pearson) with parameters “-s -w 0”. Then, from the SCCs between all combinations of non-orthologous genes, the same number of non-orthologous gene pairs as the orthologous gene pairs were random selected as a negative control. The wilcox.test function in R v3.4.3 was used to compare the two categories of SCCs.

### Reporting summary

Further information on research design is available in the Nature Research Reporting Summary linked to this article.

## Supplementary information

Supplementary Information

Description of Additional Supplementary Files

Supplementary Data 1

Supplementary Data 2

Supplementary Data 3

Supplementary Data 4

Supplementary Data 5

Supplementary Data 6

Supplementary Data 7

Supplementary Data 8

Supplementary Data 9

Supplementary Data 10

Supplementary Data 11

Supplementary Data 12

Reporting Summary

## Data Availability

Raw sequence data that support the findings of this study have been deposited in the NCBI Sequence Read Archive (SRA) under the Bioproject accession number PRJNA597497. Processed data files are available in the Gene Expression Omnibus (GEO) under accession GSE143288. Human ChIP-seq data for H3K27ac, H3K4me1, and H3K4me3 were downloaded from the Roadmap Epigenomics project (https://egg2.wustl.edu/roadmap/web_portal/processed_data.html). Mouse promoters and enhancers on the mm9 genome were downloaded from the YUE lab at Penn State (http://promoter.bx.psu.edu/ENCODE/download.html). The public ChIP-seq data of pig liver and induced pluripotent stem cells were downloaded from PRJNA152995 and PRJEB6906; the details are available in Supplementary Table 5. Source data are provided with this paper.

## Source data

Source Data

## Code availability

The scripts for *cis*-regulatory identification in this study are available at https://github.com/shzhao-lab/pig-encode.

## References

[1] The ENCODE Projects Consortium. (2012). An integrated encyclopedia of DNA elements in the human genome. Nature.

[2] Gerstein MB (2010). Integrative analysis of the Caenorhabditis elegans genome by the modENCODE project. Science.

[3] The modENCODE Consortium. (2010). Identification of functional elements and regulatory circuits by Drosophila modENCODE. Science.

[4] Yue F (2014). A comparative encyclopedia of DNA elements in the mouse genome. Nature.

[5] Shen Y (2012). A map of the cis-regulatory sequences in the mouse genome. Nature.

[6] Roadmap Epigenomics Consortium (2015). Integrative analysis of 111 reference human epigenomes. Nature.

[7] Yan S (2018). A Huntingtin knockin pig model recapitulates features of selective neurodegeneration in Huntington’s Disease. Cell.

[8] Langin M (2018). Consistent success in life-supporting porcine cardiac xenotransplantation. Nature.

[9] Renner S (2013). Permanent neonatal diabetes in INS(C94Y) transgenic pigs. Diabetes.

[10] Xie J (2019). Efficient base editing for multiple genes and loci in pigs using base editors. Nat. Commun..

[11] Luo Y (2011). High efficiency of BRCA1 knockout using rAAV-mediated gene targeting: developing a pig model for breast cancer. Transgenic Res..

[12] Wang K (2017). Cre-dependent Cas9-expressing pigs enable efficient in vivo genome editing. Genome Res..

[13] Ekser B, Li P, Cooper DKC (2017). Xenotransplantation: past, present, and future. Curr. Opin. Organ Transpl..

[14] Yang L (2015). Genome-wide inactivation of porcine endogenous retroviruses (PERVs). Science.

[15] Yue, Y. et al. Extensive mammalian germline genome engineering. *bioRxiv*10.1101/2019.12.17.876862 (2019).

[16] Young LD (1998). Survival, body weights, feed efficiency, and carcass traits of 7/8 White Composite and 1/8 Duroc, 1/8 Meishan, 1/8 Fengjing, or 1/8 Minzhu pigs. J. Anim. Sci..

[17] Wang L (2011). Animal genetic resources in China: pigs. China Agric. Ture Press.

[18] White BR (1995). Growth and body composition of Meishan and Yorkshire barrows and gilts. J. Anim. Sci..

[19] Mourot J, Kouba M (1999). Development of intra- and intermuscular adipose tissue in growing large white and Meishan pigs. Reprod. Nutr. Dev..

[20] Zhao, P. et al. Evidence of evolutionary history and selective sweeps in the genome of Meishan pig reveals its genetic and phenotypic characterization. *Gigascience* **7**, 10.1093/gigascience/giy058 (2018). 10.1093/gigascience/giy058 PMC6007440 29790964

[21] Li M (2017). Comprehensive variation discovery and recovery of missing sequence in the pig genome using multiple de novo assemblies. Genome Res..

[22] Tang Z (2007). LongSAGE analysis of skeletal muscle at three prenatal stages in Tongcheng and Landrace pigs. Genome Biol..

[23] Kojima M (2018). Differences in gene expression profiles for subcutaneous adipose, liver, and skeletal muscle tissues between Meishan and Landrace pigs with different backfat thicknesses. PLoS ONE.

[24] Xiao S (2012). Comparative epigenomic annotation of regulatory DNA. Cell.

[25] Villar D (2015). Enhancer evolution across 20 mammalian species. Cell.

[26] Servant N (2015). HiC-Pro: an optimized and flexible pipeline for Hi-C data processing. Genome Biol..

[27] Trieu T, Cheng J (2017). 3D genome structure modeling by Lorentzian objective function. Nucleic Acids Res..

[28] Trieu T, Oluwadare O, Wopata J, Cheng J (2019). GenomeFlow: a comprehensive graphical tool for modeling and analyzing 3D genome structure. Bioinformatics.

[29] Huang da W, Sherman BT, Lempicki RA (2009). Bioinformatics enrichment tools: paths toward the comprehensive functional analysis of large gene lists. Nucleic Acids Res..

[30] Huang da W, Sherman BT, Lempicki RA (2009). Systematic and integrative analysis of large gene lists using DAVID bioinformatics resources. Nat. Protoc..

[31] Yanai I (2005). Genome-wide midrange transcription profiles reveal expression level relationships in human tissue specification. Bioinformatics.

[32] Kryuchkova-Mostacci N, Robinson-Rechavi M (2017). A benchmark of gene expression tissue-specificity metrics. Brief. Bioinform..

[33] Ong CT, Corces VG (2011). Enhancer function: new insights into the regulation of tissue-specific gene expression. Nat. Rev. Genet..

[34] Heinz S (2010). Simple combinations of lineage-determining transcription factors prime cis-regulatory elements required for macrophage and B cell identities. Mol. Cell.

[35] Liu X, Yu X, Zack DJ, Zhu H, Qian J (2008). TiGER: a database for tissue-specific gene expression and regulation. BMC Bioinform..

[36] Xiao SJ, Zhang C, Zou Q, Ji ZL (2010). TiSGeD: a database for tissue-specific genes. Bioinformatics.

[37] McLean CY (2010). GREAT improves functional interpretation of cis-regulatory regions. Nat. Biotechnol..

[38] Loven J (2013). Selective inhibition of tumor oncogenes by disruption of super-enhancers. Cell.

[39] Hnisz D (2013). Super-enhancers in the control of cell identity and disease. Cell.

[40] Whyte WA (2013). Master transcription factors and mediator establish super-enhancers at key cell identity genes. Cell.

[41] Chen K (2015). Broad H3K4me3 is associated with increased transcription elongation and enhancer activity at tumor-suppressor genes. Nat. Genet..

[42] Reilly SK (2015). Evolutionary genomics. Evolutionary changes in promoter and enhancer activity during human corticogenesis. Science.

[43] Visel A, Minovitsky S, Dubchak I, Pennacchio LA (2007). VISTA Enhancer Browser–a database of tissue-specific human enhancers. Nucleic Acids Res..

[44] Crane E (2015). Condensin-driven remodelling of X chromosome topology during dosage compensation. Nature.

[45] Giorgetti L (2016). Structural organization of the inactive X chromosome in the mouse. Nature.

[46] Dixon JR (2015). Chromatin architecture reorganization during stem cell differentiation. Nature.

[47] Dixon JR (2012). Topological domains in mammalian genomes identified by analysis of chromatin interactions. Nature.

[48] Shin H (2016). TopDom: an efficient and deterministic method for identifying topological domains in genomes. Nucleic Acids Res..

[49] Nora EP (2012). Spatial partitioning of the regulatory landscape of the X-inactivation centre. Nature.

[50] Li G (2012). Extensive promoter-centered chromatin interactions provide a topological basis for transcription regulation. Cell.

[51] Rao SS (2014). A 3D map of the human genome at kilobase resolution reveals principles of chromatin looping. Cell.

[52] Edea Z (2017). Detecting selection signatures between Duroc and Duroc synthetic pig populations using high‐density SNP chip. Anim. Genet..

[53] Fontanesi L, Schiavo G, Galimberti G, Calo D, Russo V (2014). A genomewide association study for average daily gain in Italian Large White pigs. J. Anim. Sci..

[54] Meng Q (2017). Identification of growth trait related genes in a Yorkshire purebred pig population by genome-wide association studies. Asian-Australas. J. Anim. Sci..

[55] Love MI, Huber W, Anders S (2014). Moderated estimation of fold change and dispersion for RNA-seq data with DESeq2. Genome Biol..

[56] Robinson MD, McCarthy DJ, Smyth GK (2010). edgeR: a Bioconductor package for differential expression analysis of digital gene expression data. Bioinformatics.

[57] Weir BS, Cockerham CC (1984). Estimating F-statistics for the analysis of population structure. Evolution.

[58] Kuhn RM, Haussler D, Kent WJ (2013). The UCSC genome browser and associated tools. Brief. Bioinform..

[59] Franke M (2016). Formation of new chromatin domains determines pathogenicity of genomic duplications. Nature.

[60] Fudenberg G, Pollard KS (2019). Chromatin features constrain structural variation across evolutionary timescales. Proc. Natl Acad. Sci. USA.

[61] Chen J, Bardes EE, Aronow BJ, Jegga AG (2009). ToppGene Suite for gene list enrichment analysis and candidate gene prioritization. Nucleic Acids Res..

[62] Caves EM, Brandley NC, Johnsen S (2018). Visual acuity and the evolution of signals. Trends Ecol. Evol..

[63] Bult CJ (2019). Mouse Genome Database (MGD) 2019. Nucleic Acids Res..

[64] Lang MR, Lapierre LA, Frotscher M, Goldenring JR, Knapik EW (2006). Secretory COPII coat component Sec23a is essential for craniofacial chondrocyte maturation. Nat. Genet..

[65] Eivers E, McCarthy K, Glynn C, Nolan CM, Byrnes L (2004). Insulin-like growth factor (IGF) signalling is required for early dorso-anterior development of the zebrafish embryo. Int. J. Dev. Biol..

[66] Imamura S, Yabu T, Yamashita M (2012). Protective role of cell division cycle 48 (CDC48) protein against neurodegeneration via ubiquitin-proteasome system dysfunction during zebrafish development. J. Biol. Chem..

[67] Richard-Parpaillon L, Heligon C, Chesnel F, Boujard D, Philpott A (2002). The IGF pathway regulates head formation by inhibiting Wnt signaling in Xenopus. Dev. Biol..

[68] Jin Z (2016). Members of the Rusc protein family interact with Sufu and inhibit vertebrate Hedgehog signaling. Development.

[69] Square T (2015). A gene expression map of the larval Xenopus laevis head reveals developmental changes underlying the evolution of new skeletal elements. Dev. Biol..

[70] Amemiya HM, Kundaje A, Boyle AP (2019). The ENCODE blacklist: identification of problematic regions of the genome. Sci. Rep..

[71] Ernst J (2011). Mapping and analysis of chromatin state dynamics in nine human cell types. Nature.

[72] Trynka G (2013). Chromatin marks identify critical cell types for fine mapping complex trait variants. Nat. Genet..

[73] Maurano MT (2012). Systematic localization of common disease-associated variation in regulatory DNA. Science.

[74] Smemo S (2014). Obesity-associated variants within FTO form long-range functional connections with IRX3. Nature.

[75] Mifsud B (2015). Mapping long-range promoter contacts in human cells with high-resolution capture Hi-C. Nat. Genet..

[76] Jung I (2019). A compendium of promoter-centered long-range chromatin interactions in the human genome. Nat. Genet..

[77] Zhang Y (2013). Chromatin connectivity maps reveal dynamic promoter-enhancer long-range associations. Nature.

[78] Ai H (2015). Adaptation and possible ancient interspecies introgression in pigs identified by whole-genome sequencing. Nat. Genet..

[79] Buenrostro, J. D., Wu, B., Chang, H. Y. & Greenleaf, W. J. ATAC-seq: a method for assaying chromatin accessibility genome-wide. *Curr. Protoc. Mol. Biol.* **109**, 21–29 (2015). 10.1002/0471142727.mb2129s109 PMC4374986 25559105

[80] Bright, A. R. & Veenstra, G. J. C. Assay for transposase-accessible chromatin-sequencing using Xenopus embryos. *Cold Spring Harb. Protoc.* **2019**, 10.1101/pdb.prot098327 (2019). 10.1101/pdb.prot098327 30042136

[81] Lai B (2018). Trac-looping measures genome structure and chromatin accessibility. Nat. Methods.

[82] Heintzman ND (2007). Distinct and predictive chromatin signatures of transcriptional promoters and enhancers in the human genome. Nat. Genet..

[83] Trapnell C, Pachter L, Salzberg SL (2009). TopHat: discovering splice junctions with RNA-Seq. Bioinformatics.

[84] Trapnell C (2012). Differential gene and transcript expression analysis of RNA-seq experiments with TopHat and Cufflinks. Nat. Protoc..

[85] Sun L (2013). Utilizing sequence intrinsic composition to classify protein-coding and long non-coding transcripts. Nucleic Acids Res..

[86] Kang YJ (2017). CPC2: a fast and accurate coding potential calculator based on sequence intrinsic features. Nucleic Acids Res..

[87] Li B, Dewey CN (2011). RSEM: accurate transcript quantification from RNA-Seq data with or without a reference genome. BMC Bioinform..

[88] Ramirez F, Dundar F, Diehl S, Gruning BA, Manke T (2014). deepTools: a flexible platform for exploring deep-sequencing data. Nucleic Acids Res..

[89] Altschul SF, Gish W, Miller W, Myers EW, Lipman DJ (1990). Basic local alignment search tool. J. Mol. Biol..

[90] Hicks SC (2018). Smooth quantile normalization. Biostatistics.

[91] Kolde, R. pheatmap: Pretty heatmaps. https://cran.r-project.org/package=pheatmap (2019).

[92] Li H, Durbin R (2009). Fast and accurate short read alignment with Burrows-Wheeler transform. Bioinformatics.

[93] Li H (2009). The Sequence Alignment/Map format and SAMtools. Bioinformatics.

[94] Feng J, Liu T, Qin B, Zhang Y, Liu XS (2012). Identifying ChIP-seq enrichment using MACS. Nat. Protoc..

[95] Landt SG (2012). ChIP-seq guidelines and practices of the ENCODE and modENCODE consortia. Genome Res..

[96] Bolstad, B. M. & Bolstad, B. preprocessCore: A collection of pre-processing functions. (2015).

[97] Quinlan AR, Hall IM (2010). BEDTools: a flexible suite of utilities for comparing genomic features. Bioinformatics.

[98] Gupta S, Stamatoyannopoulos JA, Bailey TL, Noble WS (2007). Quantifying similarity between motifs. Genome Biol..

[99] Martin M (2011). Cutadapt removes adapter sequences from high-throughput sequencing reads. EMBnet. J..

[100] Langmead B, Salzberg SL (2012). Fast gapped-read alignment with Bowtie 2. Nat. Methods.

[101] Bentsen M (2020). ATAC-seq footprinting unravels kinetics of transcription factor binding during zygotic genome activation. Nat. Commun..

[102] Durand NC (2016). Juicer provides a one-click system for analyzing loop-resolution Hi-C experiments. Cell Syst..

[103] Robinson JT (2011). Integrative genomics viewer. Nat. Biotechnol..

[104] Imakaev M (2012). Iterative correction of Hi-C data reveals hallmarks of chromosome organization. Nat. Methods.

[105] Fu Y (2020). A gene prioritization method based on a swine multi-omics knowledgebase and a deep learning model. Commun. Biol..

[106] Kodama, Y., Shumway, M. & Leinonen, R. on behalf of the International Nucleotide Sequence Database Collaboration. The sequence read archive: explosive growth of sequencing data. *Nucleic Acids Res.* **40**, D54–D56 (2011). 10.1093/nar/gkr854 PMC3245110 22009675

[107] Bolger AM, Lohse M, Usadel B (2014). Trimmomatic: a flexible trimmer for Illumina sequence data. Bioinformatics.

[108] Danecek P (2011). The variant call format and VCFtools. Bioinformatics.

[109] The GTEx Consortium. (2015). The Genotype-Tissue Expression (GTEx) pilot analysis: multitissue gene regulation in humans. Science.

