# Supplementary material for: A compendium and comparative epigenomics analysis of cis-regulatory elements in the pig genome
Source: Nat Commun. 2021 Apr 13;12:2217. doi: 10.1038/s41467-021-22448-x (PMC8044108; doi:10.1038/s41467-021-22448-x)
Supplement: Supplementary file 1 — Supplementary Information [file 41467_2021_22448_MOESM1_ESM.pdf]

Supplementary Figure

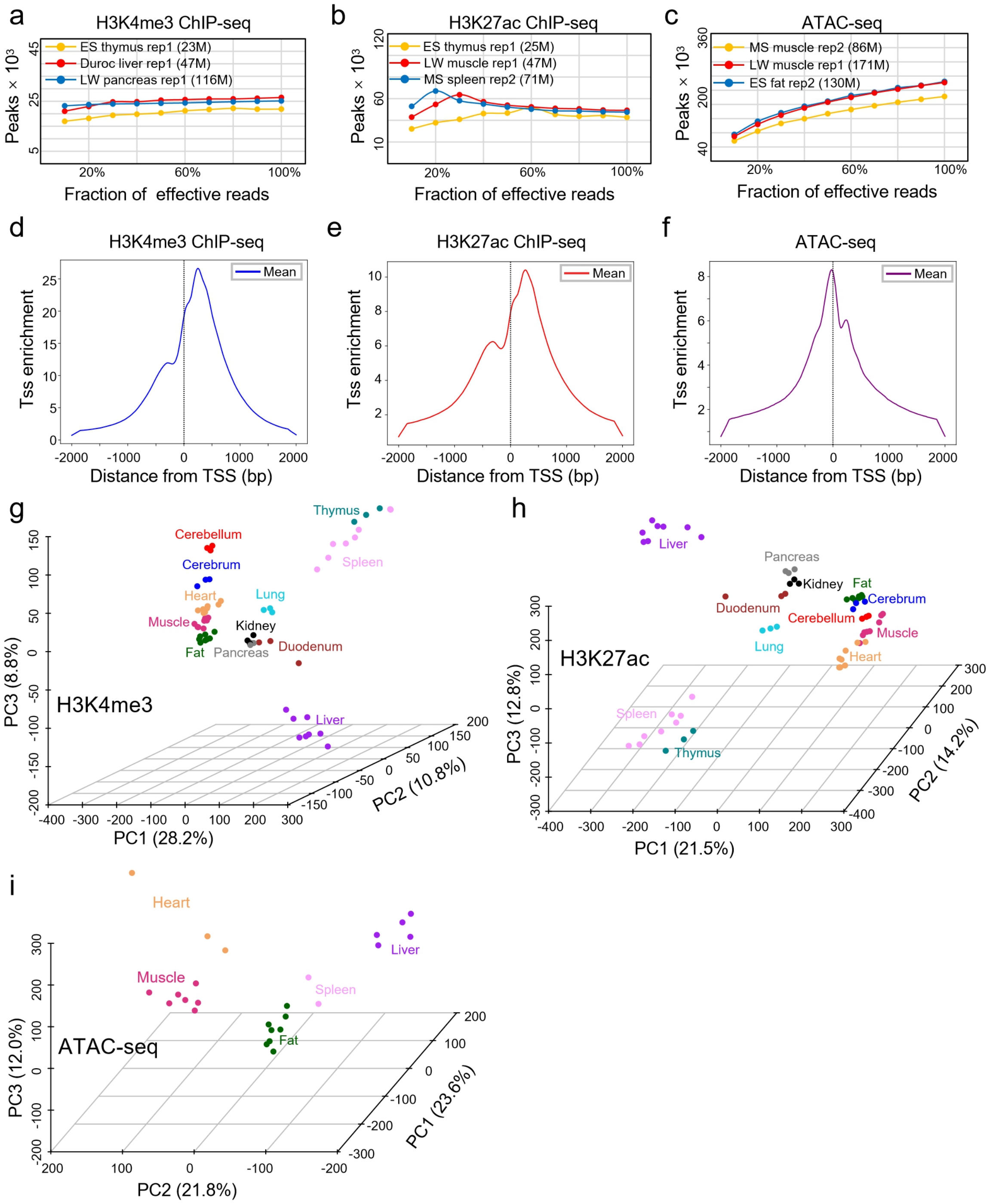

Supplementary Fig. 1 The quality control for ChIP-seq and ATAC-seq data.

MS, LW, and ES refer to Meishan, Large White, and Enshi Black pigs, respectively. Rep1 and rep2 refer to biological replicate 1 and biological replicate 2, respectively.

a–c: Plots of saturation analyses for ChIP-seq and ATAC-seq libraries. Each sample was down-sampled (10%, 20%, ..., 90%) from effective reads (which passed filtering criteria) to call narrow peaks ($P < 10^{-5}$). The number following the sample name indicates effective read count. **d–e**: TSS enrichment plots of H3K4me3 ChIP-seq, H3K27ac ChIP-seq, and ATAC-seq experiments of Duroc muscle rep1. **g–i:** PCA plots of H3K4me3 ChIP-seq, H3K27ac ChIP-seq, and ATAC-seq data.

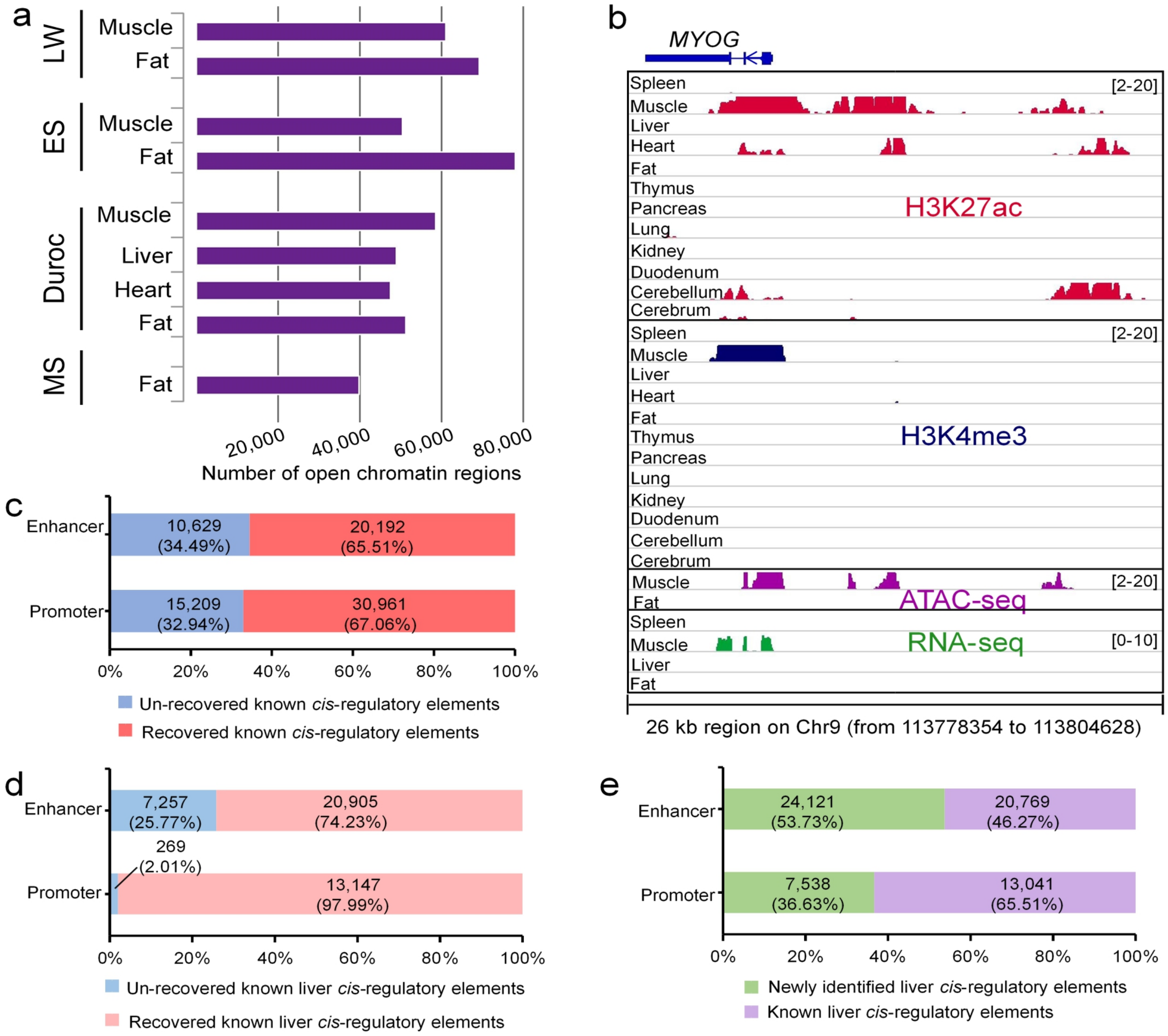

Supplementary Fig. 2 Supplementary information for the landscape for *cis*-regulatory elements of the pig genome.

MS, LW, and ES refer to Meishan, Large White, and Enshi Black pigs, respectively.

a Open chromatin landscape of the pig genome. **b** Genome browser views of ChIP-seq, ATAC-seq, and RNA-seq data for the *MYOG* gene region in various LW pig tissues. The numbers in brackets located in the ChIP-seq, ATAC-seq, and RNA-seq tracks indicate signal intensities. **c** *Cis*-regulatory elements identified from UCSC TSSs and published ChIP-seq data of pig pluripotent stem cells[1] and liver tissue[2] that were also recovered by this study (red bar). **d** *Cis*-regulatory elements identified from published ChIP-seq data of pig liver tissue[2] recovered by liver data in this study. **e** *Cis*-regulatory elements identified from liver ChIP-seq data in this study recovered by published data acquired from pig liver tissue[2].

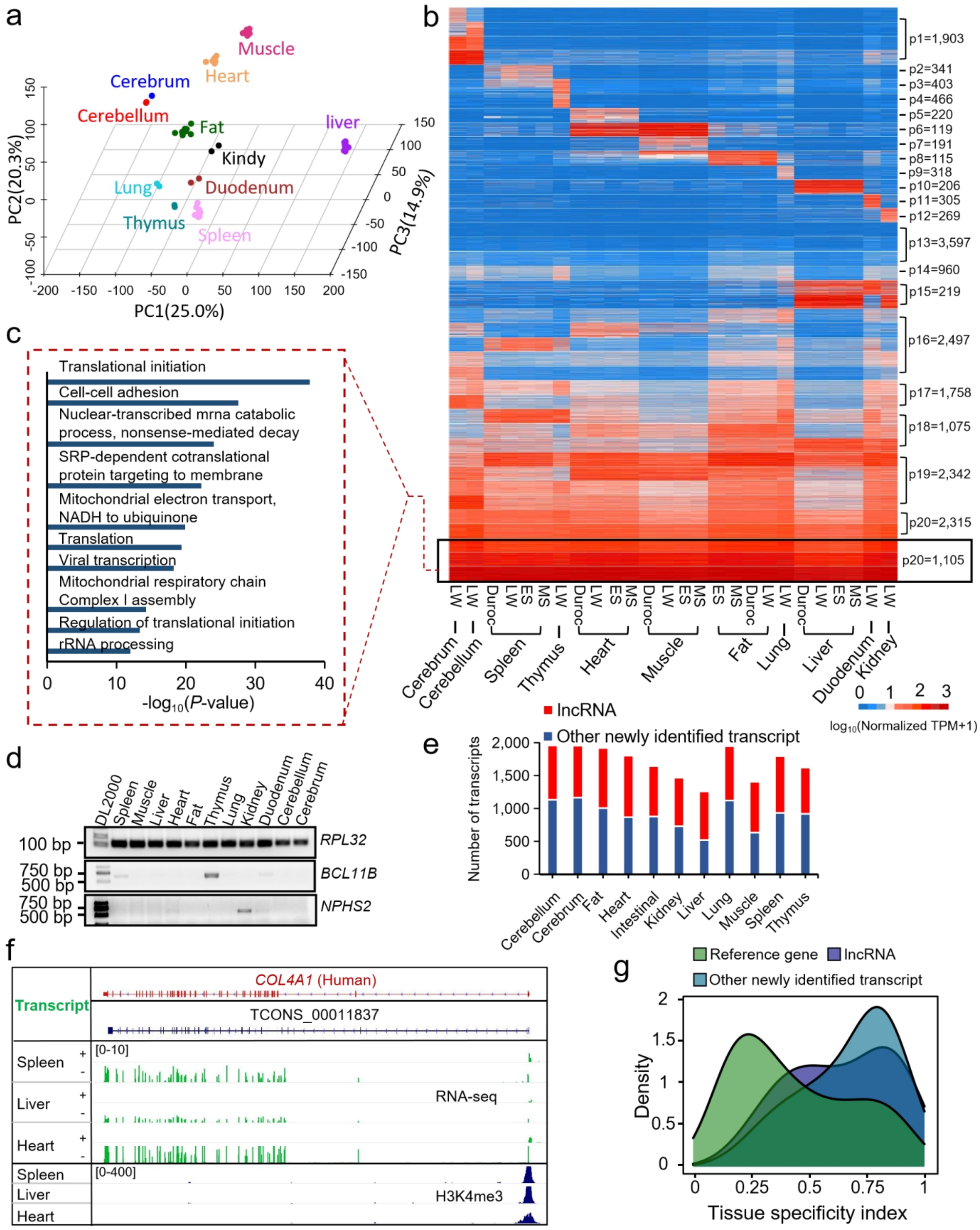

Supplementary Fig. 3 Analysis of tissue-specific genes, lncRNAs, and previously identified transcripts.
a PCA plots of RNA-seq data. **b** Overall gene expression patterns across different tissues of the four pig breeds. The p20 was split into two clusters following the limitation of gene numbers when using DAVID functional enrichment analysis. **c** GO terms of p20 cluster genes that were highly expressed in all samples. **d** Validation of tissue-specific gene expression in 11 tissues by RT-PCR. The validation was repeated twice independently with similar results and *RPL32* gene was used as a control. The PCR target fragment size of *RPL32* is 93 bp, *BCL11B* specifically expressed in thymus is 586 bp, and *NPHS2* specifically expressed in kidney is 562 bp. The original gel pictures of RT-PCR, including this figure and its repeated experiment, were provided as a source data file. **e** The number of newly identified pig lncRNAs and other transcripts detected in each tissue. **f** An example of a newly identified potential protein-coding transcript (TONS_00011837) measured by RNA-seq data and the H3K4me3 signal enriched in its promoter region in multiple tissues. The numbers in brackets located in ChIP-seq and RNA-seq tracks indicate signal intensities. **g** Distribution of tissue specificity index for reference genes, lncRNAs, and other newly identified transcripts.

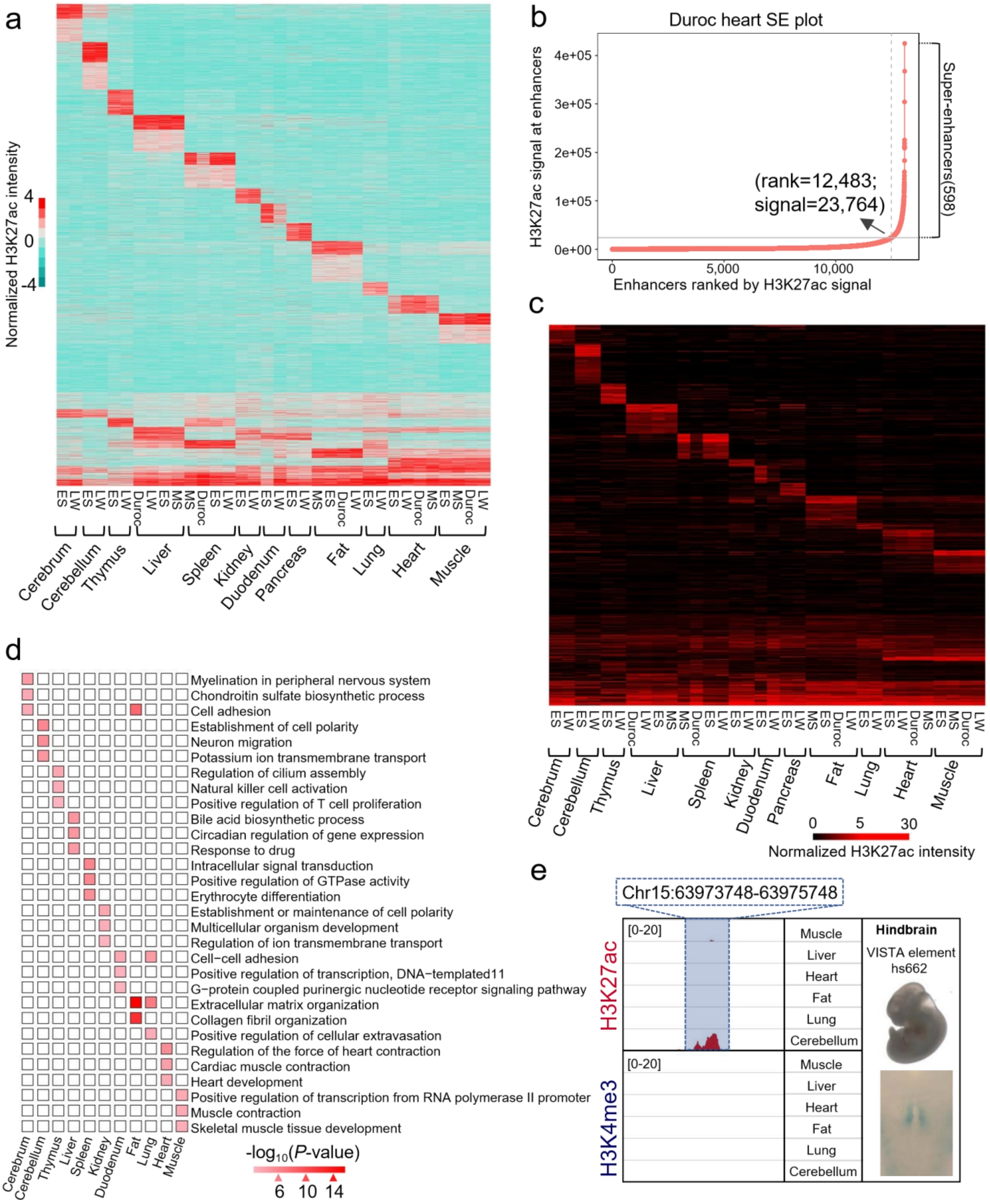

Supplementary Fig. 4 Analysis of enhancers and super-enhancers across all samples.

a Classification of enhancers based on their chromatin states (H3K27ac) among all samples of the four pig breeds. **b** Distribution of H3K27ac ChIP-Seq signals across enhancers in heart tissues of Duroc pigs. Enhancers are ranked by their input-normalized H3K27ac ChIP-seq signal. Super-enhancers (SEs) are defined as the enhancers above the curve inflection point. **c** Heatmap showing the tissue-specific patterns of super-enhancers among all samples based on normalized H3K27ac intensity. **d** GO terms enriched among tissue-specific super-enhancers. The two-side binomial statistic method without adjustment was adopted to calculate the *P*-value. **e** Tissue-specific enhancers from pig cerebellum bearing the conservation with VISTA-validated elements hs662[3]. The numbers in brackets located in the tracks of ChIP-seq indicate signal intensities.

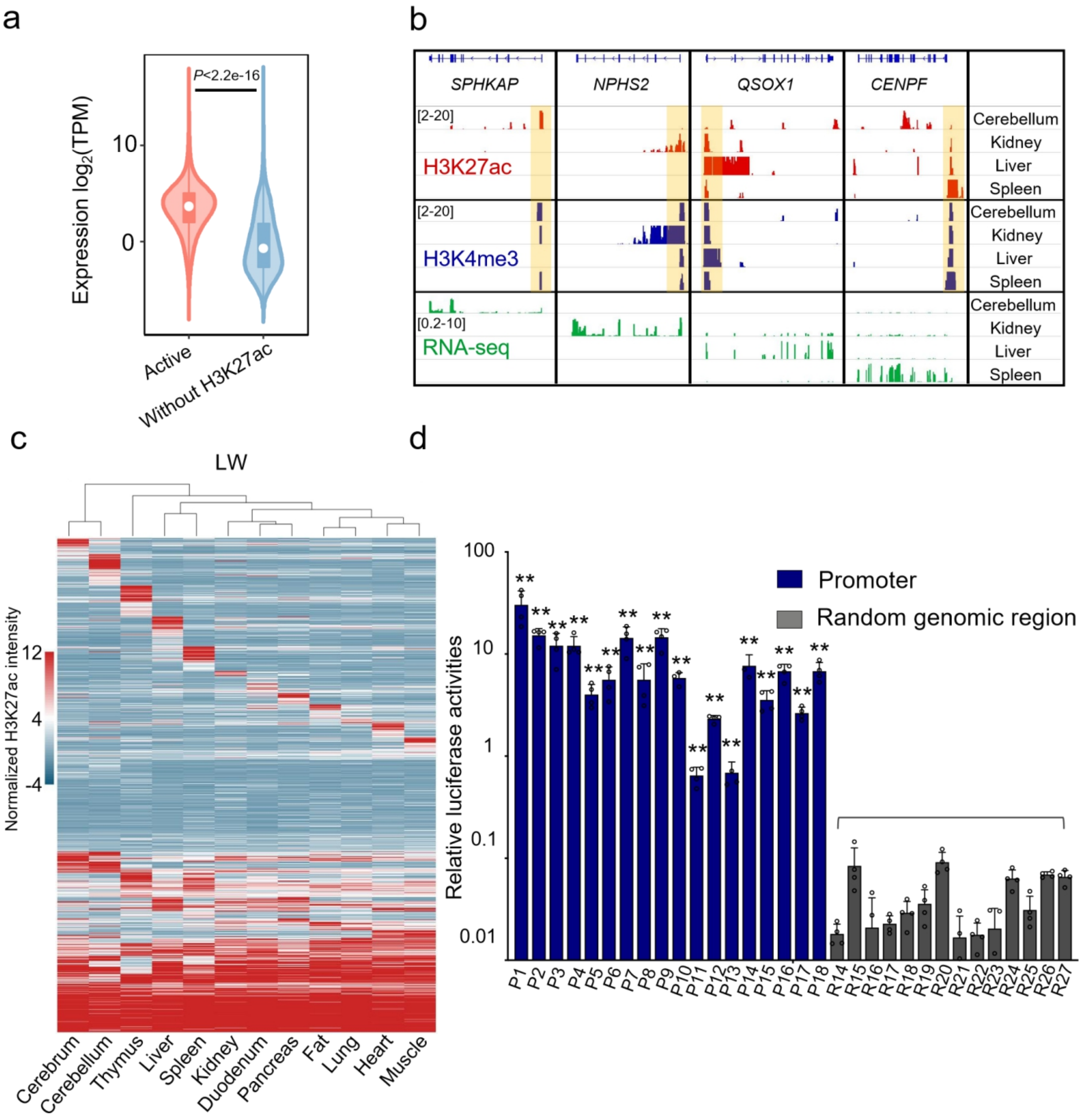

Supplementary Fig. 5 Promoter analysis of the pig genome.

a Comparison of the expression profiles of genes with active promoters (n = 318,023) to those of genes without H3K27ac promoters (n = 223,803). *P*-value was calculated by two-sided unpaired Wilcoxon test. **b** Examples of histone modification intensities (H3K27ac, H3K4me3) and gene expression profiles of active promoter-associated genes. The numbers in brackets located in the ChIP-seq and RNA-seq tracks indicate signal intensities. **c** Characteristics of active promoters based on their H3K27ac signals among Large White pig tissues. **d** Validation of promoters identified in this study using reporter assays in pig 3D4/21 cells. Data are represented as mean ± SD (n = 4). ** indicates $P < 0.01$ (1.7e-55 < P < 3.7e-27), which were calculated by two-side Student's *t*-test without multiple comparison.

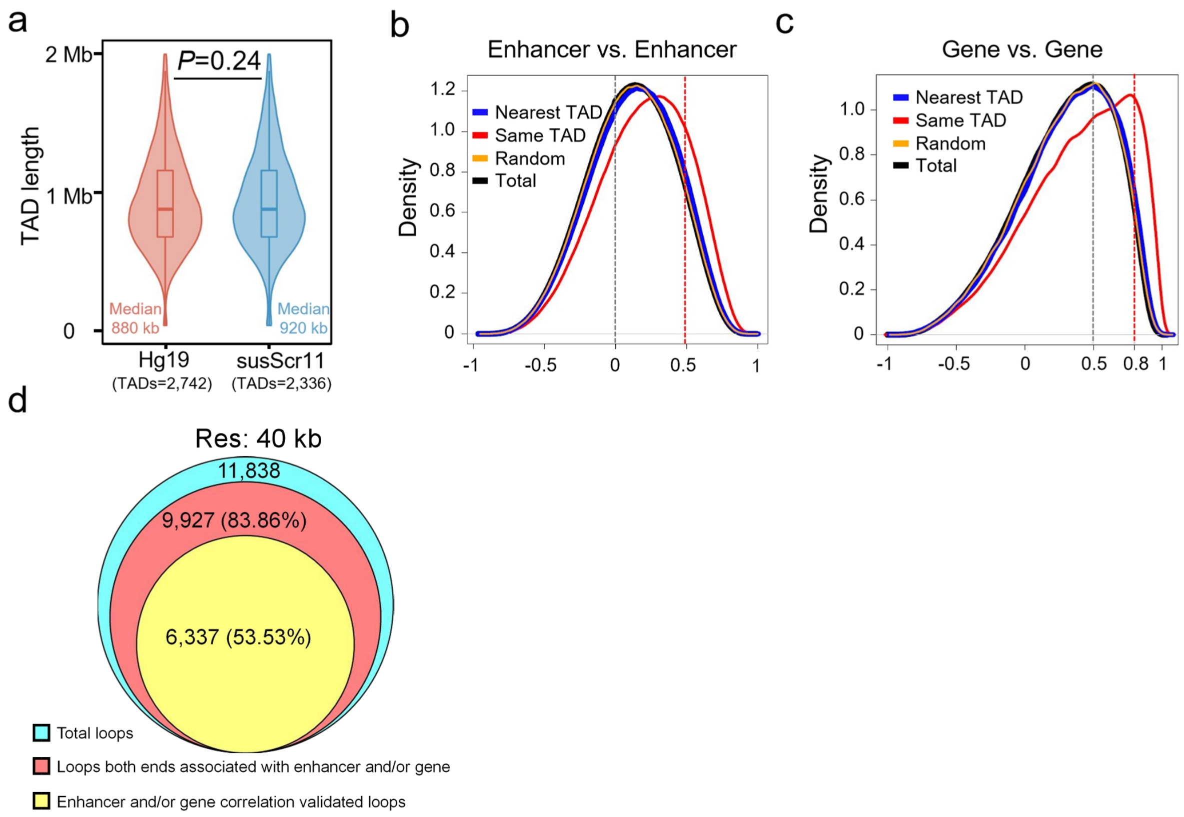

Supplementary Fig. 6 Analysis of 3D structure and regulation of *cis*-regulatory elements.

a Violin plot of TAD length in human and pig genomes (two-side unpaired Wilcoxon test). **b** Distribution of Spearman correlation coefficients between the H3K27ac intensities of enhancers (enhancer-enhancer correlations). The red dotted line indicates the estimated cutoff for significant correlation. **c** Distribution of Spearman correlation coefficients for the expression of any two genes (gene-gene correlations). The red dotted line indicates the estimated cutoff for significant correlation. The gray dotted line indicates the negative control of Spearman correlation coefficients used to set the cutoffs for significant correlation. **d** Venn diagram showing the number of total identified Hi-C loops (blue circle), loops associated with enhancers and/or genes (red circle), and loops validated by significantly correlated enhancers and/or genes (yellow circle) at 40 kb resolution (res).

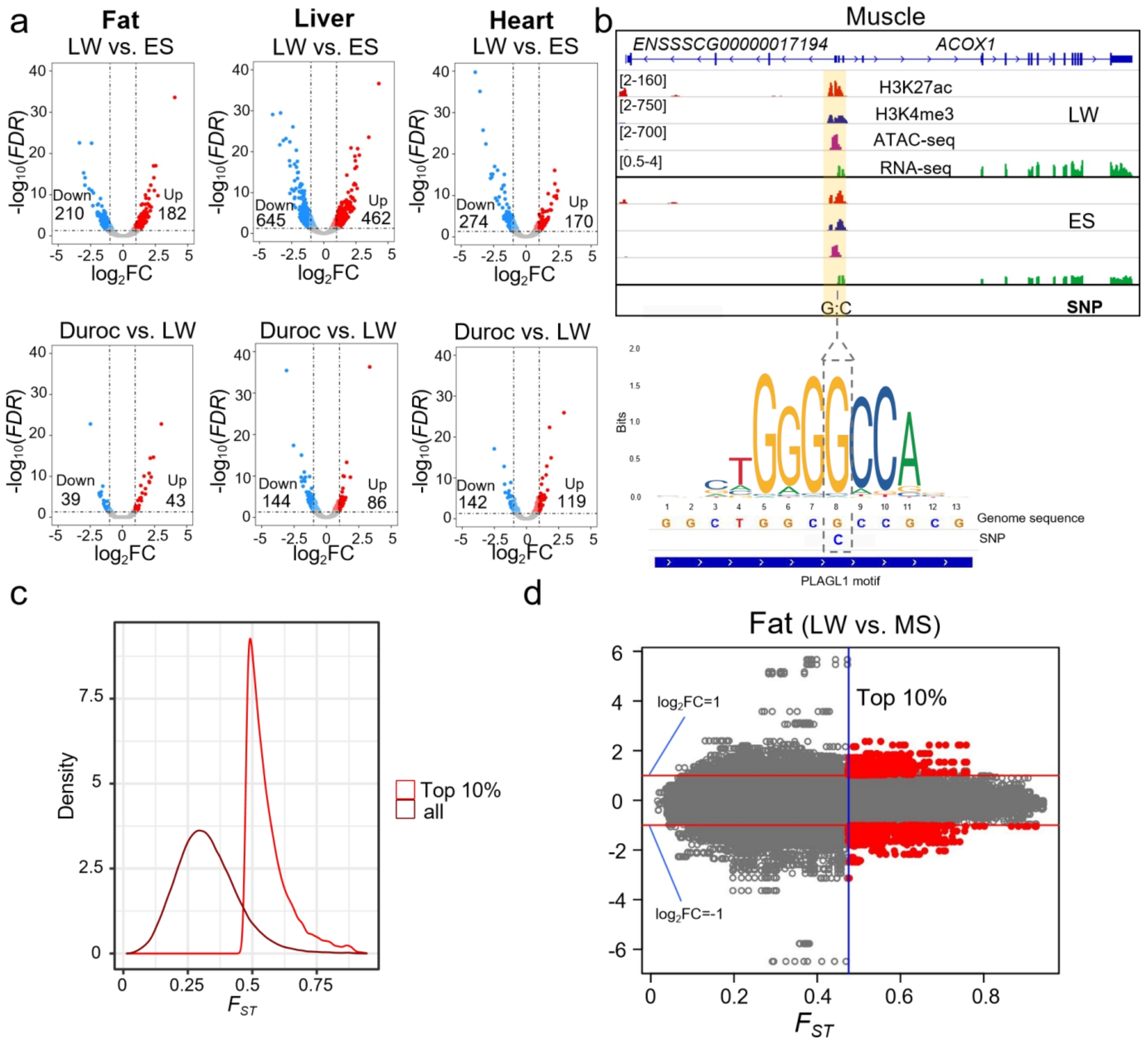

Supplementary Fig. 7 Differentially expressed genes and differences in histone intensity of *cis*-regulatory elements between pig breeds. LW and MS represent Large White and Meishan breeds respectively.

a Differentially expressed genes in LW compared with ES and Duroc compared with LW. The red dots indicate up-regulated genes, and blue dots indicate down-regulated genes. **b** An example of a G/C SNP (Chr12:5451199) with ΔAF = 0.64 between LW and ES pigs in the active promoter region corresponding to the differentially expressed *ACOX1* gene between LW and ES pigs. **c** Comparison of the distributions of top 10% and all F_{ST} values in LW compared with MS. **d** Distribution of F_{ST} values and $\log_2$FC values of H3K27ac intensities in active promoter/enhancer regions in fat tissues of LW compared with MS.

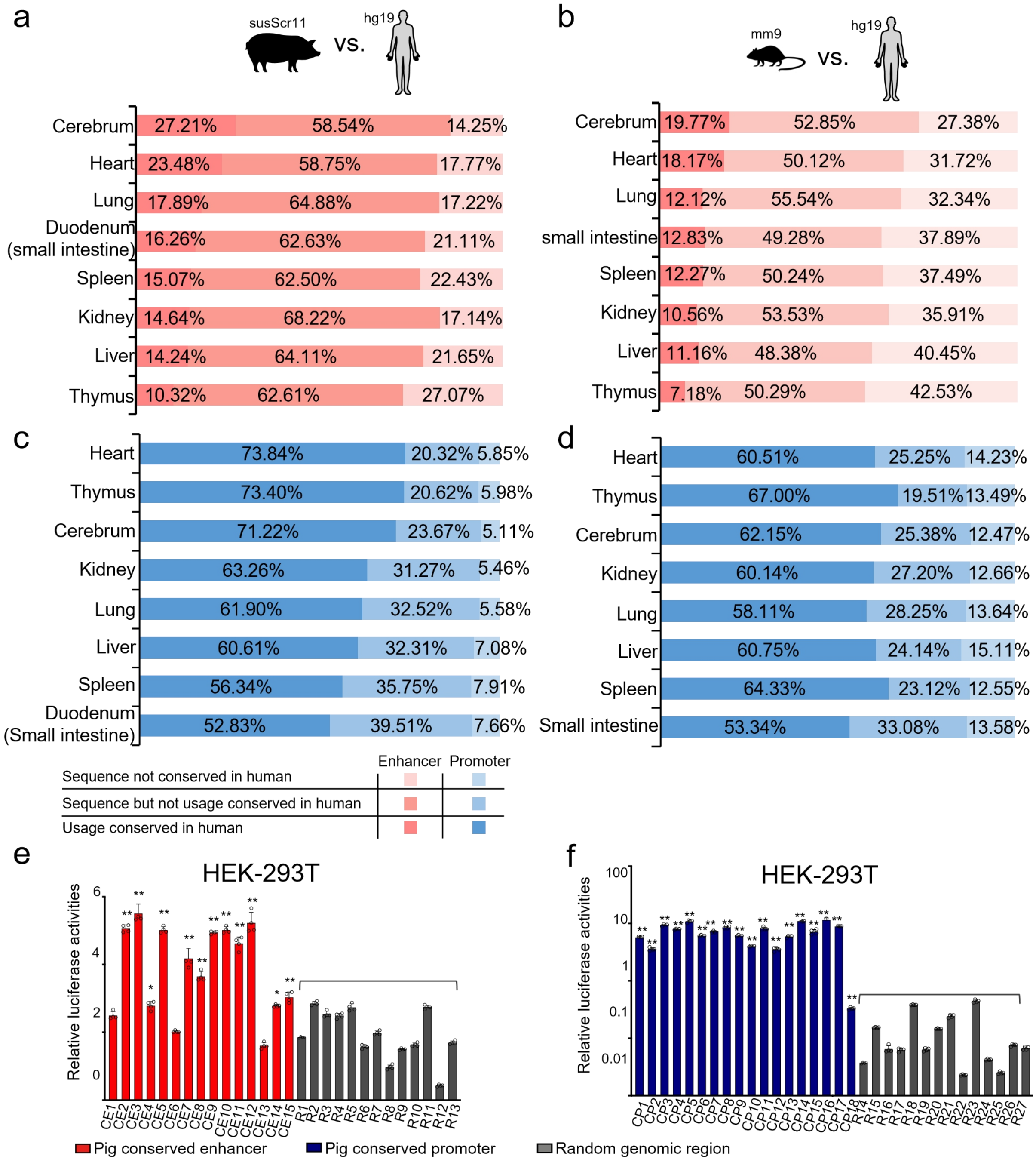

Supplementary Fig. 8. Comparison of *cis*-regulatory element conservation levels among pig, human, and mouse genomes and reporter assays for detection of pig-human conserved *cis*-regulatory element activity in human HEK-293T cells.

CP (CE) indicates pig promoter (enhancer) conserved in sequence with human genome sequence.

a–**d** Comparison of conservation levels of *cis*-regulatory elements in each tissue between pig and human (**a** and **c**) and between mouse and human (**b** and **d**). Degree of enhancer conservation is indicated with red shading (**a** and **b**), and degree of promoter conservation with blue shading **(c** and **d**). **e** Reporter assays analyzing the activity of pig enhancers conserved in sequence and usage with humans in HEK-293T cells (n = 4, 9.9e-14 < P < 0.78). The enhancers and random regions from the pig genome used for this experiment were the same as those presented in Fig. 2h. **f** Reporter assays analyzing the activities of pig promoters conserved in sequence and usage with humans in HEK-293T cells (n = 4, 1.5e-76 < P < 0.0035). The promoters and random regions from the pig genome used for this experiment were the same as those presented in Supplementary Fig. 5d.

In **e**–**f,** data are presented as means ± SD, ** indicates P < 0.01 and * indicates P < 0.05. P-values were calculated using two-side Student's *t*-test method without multiple comparison.

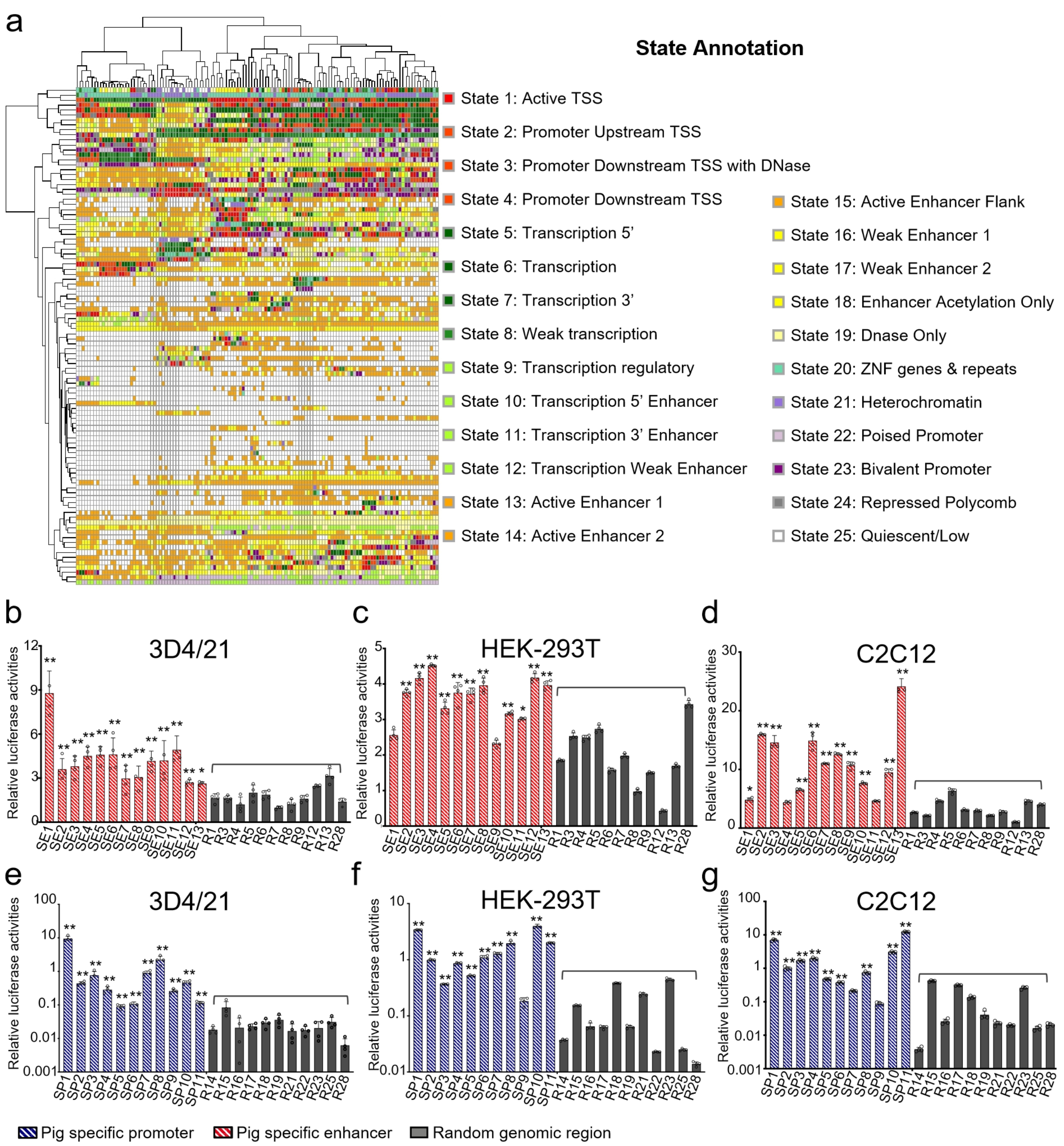

Supplementary Fig. 9 Human chromatin state annotations overlapping with pig enhancers and reporter assays of pig-specific *cis*-regulatory element activity.

SP (SE) indicates pig-specific promoter (enhancer) sequence.

a Human chromatin state annotations overlapping with pig enhancers, which were conserved in sequence but not in usage with the human genome. Each row represents one enhancer cluster, and each column corresponds to a different human tissue or cell line. Light green, orange, and yellow states correspond to human enhancers (states 9–18). **b** Reporter assays to validate pig-specific enhancer activity in pig 3D4/21 cells (n = 4, 2.4e-22 < P < 0.011). **c** Reporter assays to test the activity of pig-specific enhancers in human HEK-293T cells (n = 4, 1.1e-07 < P < 0.35). **d** Reporter assays to test the activity of pig-specific enhancers in mouse C2C12 myoblast cells (n = 4, 1.3e-30 < P < 0.58). **e** Reporter assays to validate pig-specific promoters in pig 3D4/21 cells (n = 4, 8.4e-37 < P < 7.5e-06). **f** Detection of pig-specific promoter activity in human HEK-293T cells (n = 4, 4.8e-39 < P < 0.55). **g** Detection of pig specific promoter activity in mouse C2C12 myoblast cells (n = 4, 8.0e-62 < P < 0.67).

In **b–g,** data are presented as means ± SD, ** indicates P < 0.01 and * indicates P < 0.05. P-values were calculated using two-side Student's t-test method without multiple comparison.

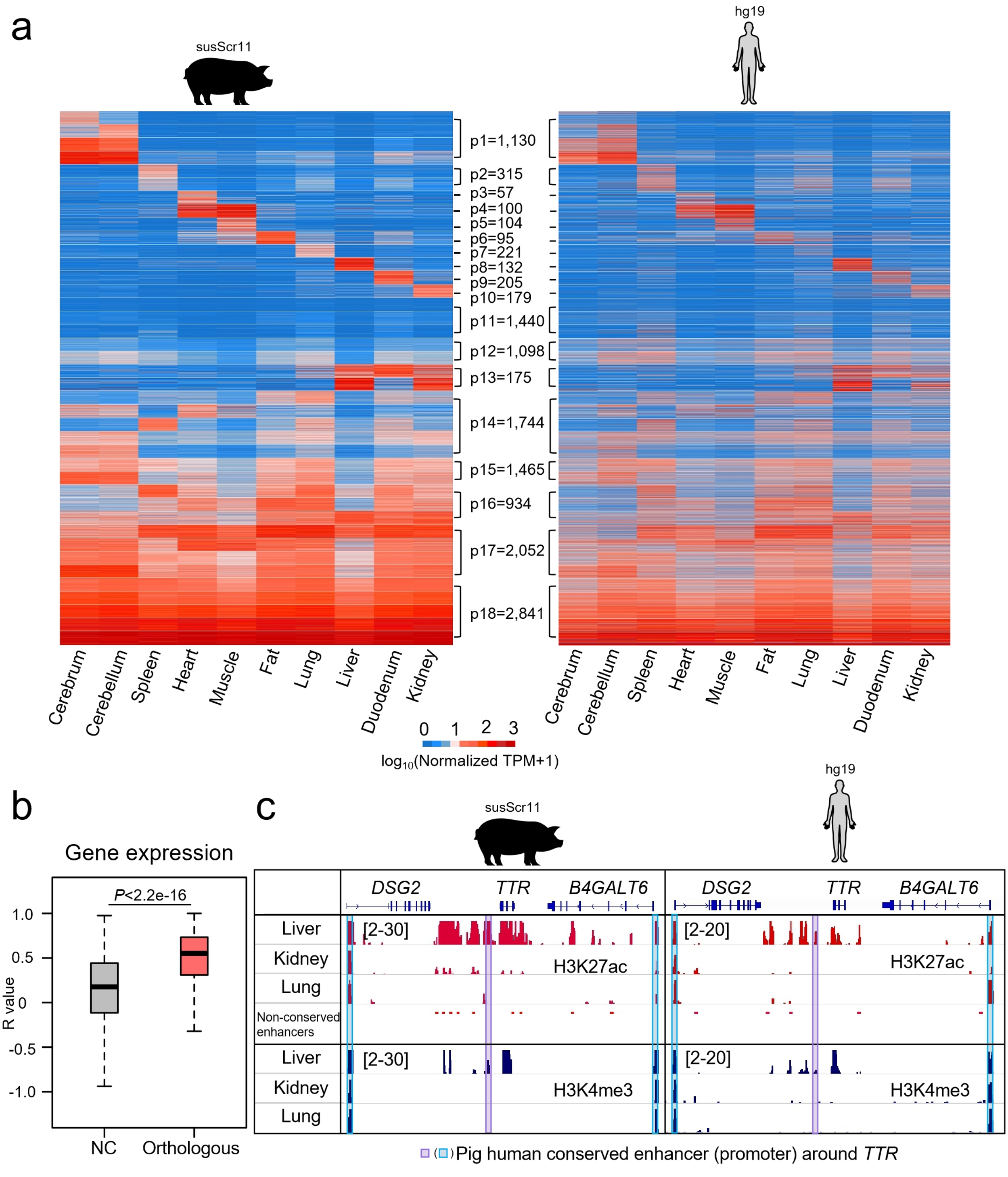

Supplementary Fig. 10 Comparison of orthologous gene expression patterns between pigs and humans.

a The expression patterns of pig and human orthologous genes. The list of human genes and their expression levels were obtained from the GTEx project[4]. **b** Spearman correlation coefficients of expression between pig-human orthologous gene pairs and non-orthologous genes in various pig and human concordant tissues. "NC" indicates correlation between non-orthologous genes pairs which were random selected from all combinations of non-orthologous genes. The bounds of boxplots represent the 25th percentile, median, and 75th percentile. The minima and maxima values of boxplots were defined after excluding outliners. The *P*-value was determined using a two-side unpaired Wilcoxon test (n = 14,287). **c** Examples of enhancer (purple shading) and promoter (blue shading) conservation between pigs and humans at the *TTR* gene locus in multiple tissues. The numbers in brackets located in the ChIP-seq tracks indicate signal intensities.

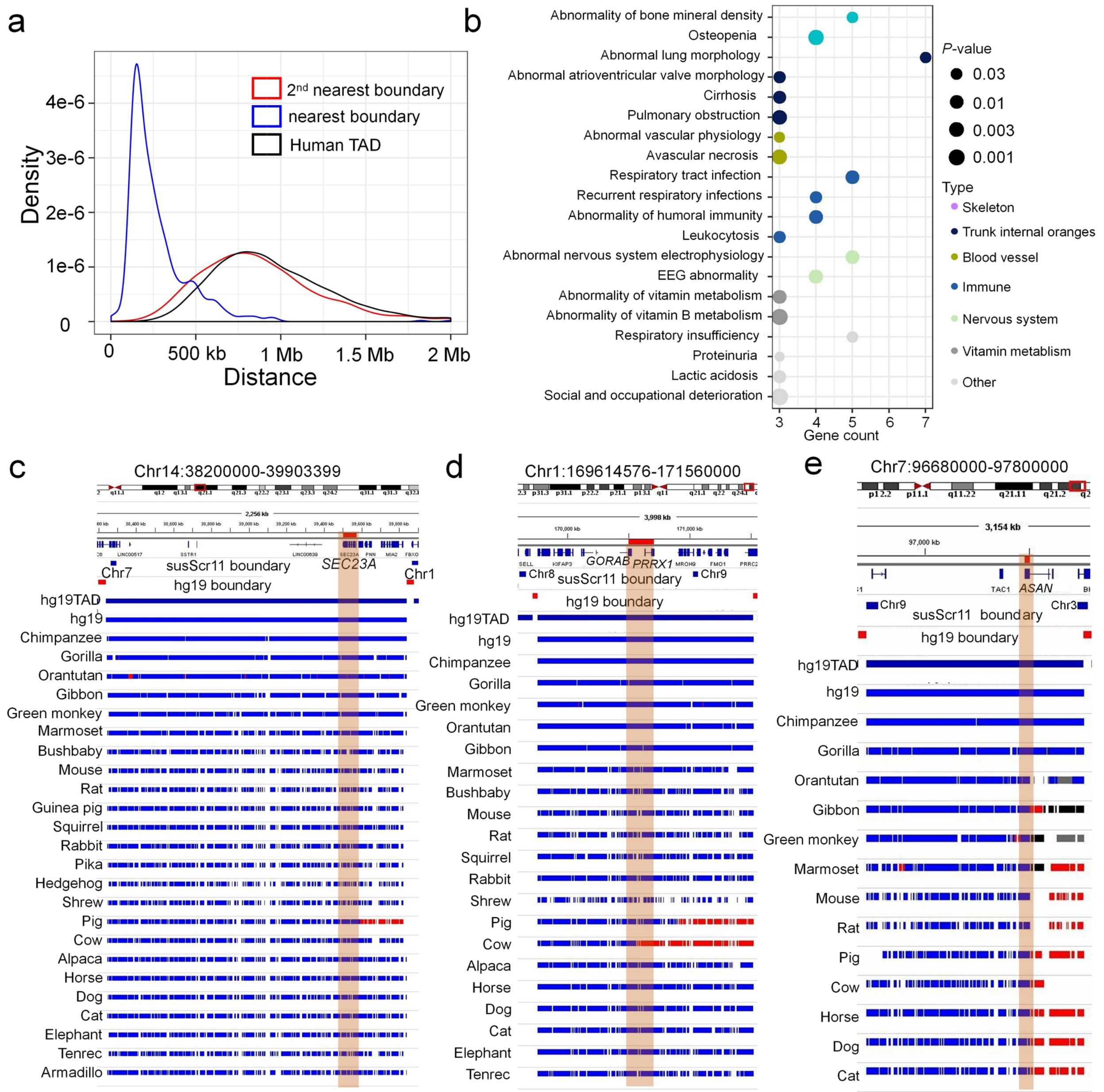

Supplementary Fig. 11 Conservation of TADs between humans and pigs.

a Distribution of span lengths of regions of human genome sequence conserved with pig TAD boundaries and their nearest and second nearest human boundaries. Here, 481 regions of human genome sequence conserved with pig TAD boundaries were located within 500 kb of their nearest human boundaries (blue line). Moreover, the human TAD boundaries contained no other conserved pig TAD boundaries. The distribution of span lengths of these 481 human genome regions and their second nearest human boundaries (red line) were almost the same as the distribution of human TAD (black line) lengths. Therefore, we further defined these 481 pig boundaries, which included 20.71% of total pig boundaries, as human boundaries with conserved usage. **b** Human phenotype enrichment of genes from 14 randomly selected pig/human conserved TADs. TADs for which both boundaries were conserved between pigs and humans were defined as conserved TADs. The top 20 HPO enrichment terms are shown. The *P*-values were calculated using two-side hypergeometric test without adjustments. **c**–**e** Visualization of chromosome rearrangements across different mammalian species. Different colors in a track for a given species indicate that the human genome aligns to a different chromosome. The grey color in different tracks represents ≥1 chromosome. The shading indicates genes associated with human head and face phenotypes.

Supplementary Tables

Supplementary Table 1 | Statistical analysis of mapped reads for ATAC-seq experiments

Breed	Tissue	Mitochondrial reads ratio[a]		Unique mapped reads		Filtered reads[b]	
		Replicate 1	Replicate 2	Replicate 1	Replicate 2	Replicate 1	Replicate 2
Duroc	Spleen	6.35%		184,047,889		140,298,018	
	Muscle	9.90%	5.51%	157,117,457	153,078,250	106,201,610	104,745,838
	Liver	10.53%	15.41%	208,656,821	144,553,846	152,611,172	96,178,914
	Heart	7.62%	17.39%	135,926,021	154,472,094	76,954,738	86,670,400
	Fat	2.37%	2.75%	190,432,496	182,865,457	147,714,734	138,913,556
ES	Spleen	2.95%		209,204,772		170,024,902	
	Muscle	10.15%	9.75%	212,747,840	113,930,207	157,841,918	76,568,718
	Liver		19.90%		181,753,047		133,772,530
	Heart	9.28%		179,557,321		101,088,186	
	Fat	4.21%	3.02%	191,023,405	165,961,715	148,498,100	130,163,628
LW	Muscle	6.29%	5.85%	229,254,887	151,244,604	171,563,784	93,519,080
	Liver	13.83%		143,560,489		105,078,240	
	Fat	3.35%	2.20%	199,002,806	191,772,289	150,357,324	146,041,392
MS	Muscle		10.43%		127,857,937		86,049,704
	Liver		8.77%		346,845,338		252,024,694
	Fat	2.16%	2.58%	142,385,087	137,138,574	119,645,566	104,231,824

[a]Percent of mitochondrial mapped reads

[b]The mapped reads that removed low MAPQ reads (<25), unmapped reads, mate unmapped reads, not primary alignments, reads failing platform, duplicates, and mitochondrial reads.

Supplementary Table 2 | Quality control values for ATAC-seq experiments

Breed	Tissue	Replicate	NSC[a]	RSC[b]	FRiP[c]	TSS enrichment score
Duroc	Spleen	1	1.38	0.92	0.34	11.76
	Muscle	1	1.24	1.03	0.31	8.31
	Muscle	2	1.17	1.25	0.26	8.14
	Liver	1	1.26	0.96	0.30	7.77
	Liver	2	1.15	1.07	0.23	6.26
	Heart	1	1.10	1.24	0.25	5.46
	Heart	2	1.25	1.07	0.28	7.36
	Fat	1	1.16	1.04	0.28	8.81
	Fat	2	1.08	1.03	0.20	6.99
ES	Spleen	1	1.20	1.00	0.34	11.03
	Muscle	1	1.19	1.08	0.30	10.09
	Muscle	2	1.07	0.90	0.21	6.96
	Liver	2	1.44	0.80	0.35	11.92
	Heart	1	1.19	1.47	0.38	6.91
	Fat	1	1.30	1.15	0.37	12.63
	Fat	2	1.39	1.02	0.40	14.26
LW	Muscle	1	1.16	1.08	0.29	9.84
	Muscle	2	1.18	1.06	0.28	9.90
	Liver	1	1.15	0.89	0.23	8.43
	Fat	1	1.31	1.00	0.35	12.19
	Fat	2	1.38	1.00	0.41	14.51
MS	Muscle	2	1.31	1.10	0.34	10.69
	Liver	2	1.22	1.06	0.29	8.82
	Fat	1	1.07	1.03	0.19	7.99
	Fat	2	1.26	1.02	0.29	12.99

[a]Normalized strand cross-correlation coefficient.

[b]Relative strand cross-correlation coefficient.

[c]Fraction of all mapped reads that fall into the called peak regions.

Supplementary Table 3 | Correlation for ChIP-seq and ATAC-seq experiments

Breed	Tissue	H3K27ac	H3K4me3	ATAC-seq
Duroc	Spleen	0.97	0.99	
	Muscle	0.98	0.95	0.99
	Liver	0.98	0.99	0.99
	Heart	0.98	0.98	0.99
	Fat	0.92	0.83	0.99
MS	Spleen	0.95	0.98	
	Muscle	0.95	0.99	
	Liver	0.97	0.99	
	Heart	0.96	0.99	
	Fat	0.96	0.99	0.85
LW	Spleen	0.94	1.00	
	Muscle	0.97	0.99	0.99
	Liver	0.97	0.99	
	Heart	0.98	0.98	
	Fat	0.96	0.94	0.96
	Thymus	0.98	0.97	
	Pancreas	0.97	0.99	
	Lung	0.98	0.99	
	Kidney	0.98	0.99	
	Duodenum	0.98	0.99	
	Cerebellum	0.97	0.98	
	Cerebrum	0.94	0.96	
ES	Spleen	0.95	0.99	
	Muscle	0.96	0.87	0.98
	Liver	0.96	0.99	
	Heart	0.98	0.99	
	Fat	0.97	0.96	0.98

Supplementary Table 4 | The number of identified *cis*-regulatory elements mapped in susScr3 reference genome assembly

Name	Non-redundant *cis*-regulatory sequences	Putative promoters	Potential enhancers	Open chromatin regions
Number	208,591	35,761	138,282	131,002

Supplementary Table 5 | The download ChIP-seq data from ips cells and liver tissue of pig

Accession number	ChIP-seq	Cell type/Tissue
SRR414965	H3K27ac	pig induced pluripotent stem cells
SRR414970	H3K4me3	pig induced pluripotent stem cells
SRR414976	IgG	pig induced pluripotent stem cells
ERR572292	H3K4me3	pig liver
ERR572197	H3K4me3	pig liver
ERR572167	H3K4me3	pig liver
ERR572204	H3K27ac	pig liver
ERR572261	H3K27ac	pig liver
ERR572214	H3K27ac	pig liver
ERR572157	Input	pig liver
ERR572294	Input	pig liver
ERR572138	Input	pig liver

Supplementary Table 6 | The number of unique mapped reads, RNA Integrity Number, and correlation for RNA-seq experiments

Breed	Tissue	Replicate 1	RIN 1	Replicate 2	RIN 2	Correlation
Duroc	Spleen	45,023,496	8.10	93,802,182	8.70	0.97
	Muscle	79,604,762	8.30	88,160,704	8.10	0.98
	Liver	87,389,448	8.30	78,580,372	8.60	0.98
	Heart	91,180,342	7.50	89,983,492	8.30	0.98
	Fat	69,033,592	7.00	68,777,234	7.90	0.97
MS	Spleen	59,136,646	9.00	57,996,184	7.90	0.97
	Muscle	104,227,268	8.00	60,398,850	6.90	0.97
	Liver	97,352,710	7.90	67,210,322	7.60	0.98
	Heart	111,287,750	8.40	59,769,844	7.40	0.98
	Fat	59,136,646	7.30	57,996,184	6.10	0.97
LW	Spleen	83,823,602	7.90	68,226,530	8.20	0.97
	Muscle	78,131,960	7.10	100,740,776	7.40	0.98
	Liver	69,487,624	7.80	81,921,330	7.80	0.98
	Heart	75,708,664	7.40	68,903,592	7.30	0.98
	Fat	77,846,906	7.80	81,205,980	6.90	0.97
	Thymus	72,823,908	7.50	83,121,878	7.20	0.98
	Lung	72,735,404	8.00	72,285,574	7.30	0.98
	Kidney	77,902,954	7.90	85,964,246	8.50	0.97
	Duodenum	65,360,368	7.30	65,732,460	7.90	0.96
	Cerebellum	67,119,442	7.70	90,329,414	8.30	0.98
	Cerebrum	94,593,442	7.70	81,014,810	6.90	0.99
ES	Spleen	67,962,716	7.90	69,763,726	8.00	0.98
	Muscle	77,904,948	7.50	62,523,866	7.50	0.98
	Liver	70,814,064	7.50	52,593,802	7.80	0.98
	Heart	81,467,506	7.70	69,053,384	7.80	0.97
	Fat	66,203,036	6.80	70,280,076	7.20	0.98

RIN: RNA Integrity Number.

Supplementary Table 7 | The information of RT-PCR primers

	Sense	Antisense	Product length (bp)
RPL32	TGGAAGAGACGTTGTGAGCAA	CGGAAGTTTCTGGTACACAATGTAA	93
MYOG	GAAAACTACCTGCCCGTCCA	GGGCATGGTTTCATCTGGGA	612
ANGPTL3	CAGCAAGACAACAGCATCAGA	TCCATCAAGCCTCCCAAAACC	420
UMOD	TTTGCTGGCAACTACGACCT	GCAGACCCATCCAAGTTCCA	470
RBP2	TTTGCCACCCGTAAGATCGC	CTCCCAAAGCCAGCAGAAAAC	395
BCL11B	TCCCAGAGGGAGCTCATCAC	ACAACTGACACTGGCATCCAA	586
NPHS2	TTCATCATCGTGACGTTCCCT	TTTTCCCCCTCCGCAGCAAT	562

Supplementary Table 8 | List of enriched known motifs from HOMER in tissue-specific enhancers

Tissue	Transcription factors
Cerebrum	SOX (SOX17, SOX2, SOX4, SOX6), SOX9, XBP1, MEF2D, HIC1, ETS (ERG, ETV2), RFX2
Cerebellum	NEUROD1, MEF2 (MEF2D, MEF2C, MEF2A), ATOH1, RORA, HIC1, NF1
Thymus	ETS (EHF, ELF1, ELF4, ELK1, ERG, ETS1, ETV1, ETV2, ETV4, FLI1), NEUROD1
Liver	FOX (FOXA1, FOXA2, FOXA3, FOXK1, FOXK2, FOXO3, FOXP1), HLF, HNF4A, NF1
Spleen	IRF (IRF1, IRF2, IRF3, IRF8), ETS (ELF4, ERG, ETS1, ETV1, ETV2, FLI1), GATA (GATA1, GATA2, GATA3, GATA6)
Kidney	TEAD (TEAD1, TEAD2, TEAD3, TEAD4), ETS (ERG, ETS1, ETV2), FOX (FOXK1, FOXK2, FOXO3, FOXP1), BCL6, FOXO1, NF1
Duodenum	HNF4A, AP1 (ATF3, BATF, FOSL2, JUNB), ETS (EHF, ELF3, ELK1, ERG, ETS1, ETV1, ETV2, ETV4, FLI1), GATA (GATA1, GATA2, GATA3, GATA4, GATA6), FOXA2, FOXO1, IRF2, NF1
Pancreas	FOX (FOXA1, FOXA2, FOXA3, FOXF1, FOXK1, FOXK2, FOXM1, FOXO3, FOXP1), FOXO1, ESRRB, MEF2D, NF1
Lung	TEAD (TEAD1, TEAD2, TEAD3, TEAD4), ETS (EHF, ELF1, ELF3, ELF4, ELK1, ERG, ETS1, ETV1, ETV2, ETV4, FLI1), FOX (FOXA1, FOXA2, FOXF1, FOXK1, FOXK2, FOXO3, FOXP1), FOXO1, BCL6, AP1 (ATF3, BATF), MEF2D, NF1, SOX2, STAT4
Fat	AP1 (FOSL2, JUNB, ATF3, BATF), FOXO1, NF1, BCL6, ETS (ERG, ETV2, ETV4, STAT4)
Heart	MEF2 (MEF2A, MEF2C, MEF2D), GATA (GATA6, GATA4, GATA2), MITF, NF1, FOXK1, ESRRB, MEIS1
Muscle	MEF2 (MEF2D, MEF2C, MEF2A), FOXO1

Tissue-specific transcript factors based on the published studies[5-7]

Supplementary Table 9 | The number of super-enhancers identified in each tissue from the four pig breeds

	Fat	Heart	Liver	Muscle	Spleen	Cerebrum	Cerebellum	Duodenum	Kidney	Lung	Pancreas	Thymus
ES	527	680	922	1,101	916	817	925	1,306	730	1,040	859	414
LW	879	699	878	980	817	773	1,069	600	865	1,045	987	437
Duroc	468	598	1,306	942	1,006							
MS	417	833	1,208	698	539							

Supplementary Table 10 | The number of broad H3K4me3 peaks and active promoters identified in each tissue from the four pig breeds

		Fat	Heart	Liver	Muscle	Spleen	Cerebrum	Cerebellum	Duodenum	Kidney	Lung	Pancreas	Thymus
Broad H3K4me3	ES	1,457	1,261	1,491	418	1,616	1,301	1,258	1,627	1,071	963	1,288	653
	LW	1,899	738	977	940	1,389	1,828	1,448	1,885	1,567	1,226	1,677	1,028
	Duroc	1,779	1,236	1,342	1,200	1,788							
	MS	1,435	1,279	1,263	1,219	1,604							
Active promoter	ES	18,008	18,177	17,896	16,591	16,982	19,222	18,052	19,437	19,111	18,861	18,706	14,144
	LW	18,889	16,559	17,128	16,245	15,886	19,816	17,128	20,138	18,639	17,959	18,556	13,971
	Duroc	17,979	16,523	17,376	17,270	17,478							
	MS	16,765	17,487	17,796	17,418	17,732							

Supplementary Table 11 | Statistics of the different 10% quantile F_{ST} regions associated differential *cis*-regulatory elements

	Total	Overlap by promoter/enhancer		Overlap by differential promoter/enhancer	
Top 10% F_{ST}	1,126	1,040	92.36%	535	51.44%
Middle 10% F_{ST}	2,928	2,648	90.44%	1,214	45.85%
Bottom 10% F_{ST}	983	886	90.13%	380	42.89%

Supplementary Table 12 | Statistics of the top 10% F_{ST} regions associated significantly differential expression genes in five tissues

Tissue	Total DE genes	Top 10% F_{ST} with differential promoter/enhancer	Differential promoter/enhancer	Sum	
Fat	250	22	60	82	32.80%
Heart	390	41	94	135	34.62%
Liver	551	68	93	161	29.22%
Muscle	352	68	109	177	50.28%
Spleen	199	15	21	36	18.09%

Supplementary Table 13 | The number of conserved *cis*-regulatory elements between pigs, humans, and mice

minMatch = 0.5	susScr11 (9 tissues)[a]			hg19 (11 tissues)[b]		mm9 (9 tissues)[c]		
Length = 1 kb	Enhancer	Promoter	Random	Enhancer	Promoter	Enhancer	Promoter	Random
total	136,319	34,289	10,000	159,977	74,066	156,548	32,849	10,000
Sequence conserved	105,414	30,498	6,134	-	-	100,564	27,216	5,863
	77.32%	88.94%	61.34%			64.23%	82.85%	58.63%
Usage conserved	31,973	18,202		-	-	30,190	15,507	
	23.45%	53.08%				19.28%	47.21%	

[a] The 9 tissues from pig were including heart, liver, spleen, kidney, lung, thymus, duodenum, cerebrum, and cerebellum.

[b] The 9 tissues from mouse were including heart, liver, spleen, kidney, lung, thymus, intestine, cerebrum, and cerebellum.

[c] The 11 tissues of human were including heart, liver, spleen, kidney, lung, thymus, intestine, brain, fat, skeletal muscle, and pancreas.

References

1 Xiao, S. *et al.* Comparative epigenomic annotation of regulatory DNA. *Cell* **149**, 1381-1392, doi:10.1016/j.cell.2012.04.029 (2012).

2 Villar, D. *et al.* Enhancer evolution across 20 mammalian species. *Cell* **160**, 554-566, doi:10.1016/j.cell.2015.01.006 (2015).

3 Visel, A., Minovitsky, S., Dubchak, I. & Pennacchio, L. A. VISTA Enhancer Browser--a database of tissue-specific human enhancers. *Nucleic Acids Res* **35**, D88-92, doi:10.1093/nar/gkl822 (2007).

4 The GTEx Consortium *et al*. The Genotype-Tissue Expression (GTEx) pilot analysis: multitissue gene regulation in humans. *Science* **348**, 648-660, doi:10.1126/science.1262110 (2015).

5 Liu, X., Yu, X., Zack, D. J., Zhu, H. & Qian, J. TiGER: A database for tissue-specific gene expression and regulation. *BMC Bioinformatics* **9**, 271, doi:10.1186/1471-2105-9-271 (2008).

6 Shen, Y. *et al.* A map of the cis-regulatory sequences in the mouse genome. *Nature* **488**, 116-120, doi:10.1038/nature11243 (2012).

7 Xiao, S.-J., Zhang, C., Zou, Q. & Ji, Z.-L. TiSGeD: a database for tissue-specific genes. *Bioinformatics* **26**, 1273-1275, doi:10.1093/bioinformatics/btq109 (2010).
