# Supplementary material for: A compendium and comparative epigenomics analysis of cis-regulatory elements in the pig genome
Source: Nat Commun. 2021 Apr 13;12:2217. doi: 10.1038/s41467-021-22448-x (PMC8044108; doi:10.1038/s41467-021-22448-x)
Supplement: Supplementary file 2 — Description of Additional Supplementary Files [file 41467_2021_22448_MOESM2_ESM.pdf]

Description of Additional Supplementary Files

File Name: Supplementary Data 1
Description: **The number of unique mapped reads and filtered reads for ChIP-seq experiments**. "Filtered reads" indicates the mapped reads that removed low MAPQ reads (< 25), unmapped reads, mate unmapped reads, not primary alignments, reads failing platform, and duplicates.

File Name: Supplementary Data 2
Description: **Quality control values for ChIP-seq experiments.** "NSC" represents normalized strand cross-correlation coefficient. "RSC" represents relative strand cross-correlation coefficient. "FRiP" represents fraction of all mapped reads that fall into the called peak regions.

File Name: Supplementary Data 3
Description: **The tissue-specific expressed genes identified in pig tissues.**

File Name: Supplementary Data 4
Description: **The top 5 GO terms of tissue-specific genes relevant to function of corresponding tissues.** The *P*-values were calculated by a two-side modified fisher exact test without adjustments.

File Name: Supplementary Data 5
Description: **The information of newly identified transcripts in pig.**

File Name: Supplementary Data 6
Description: **The tissue-specific expressed enhancers identified in pig tissues (susScr11.1 reference genome assembly).**

File Name: Supplementary Data 7
Description: **The top 5 GO terms of tissue-specific enhancers relevant to function of corresponding tissues.** The twoside binomial statistic method without adjustment was adopted to calculate the *P*-value.

File Name: Supplementary Data 8
Description: **The enhancers conserved with VISTA enhancers.**

File Name: Supplementary Data 9
Description: **Significantly differential expressed genes from four pig breeds.** The two-side Wald statistics test with FDR adjustment was adopted to identify the significantly differential expression genes.

File Name: Supplementary Data 10
Description: **Pig-human rearranged TAD of head and face phenotype related gene, Related to Figures 5G and 5H.**

File Name: Supplementary Data 11
Description: **The HPO of human genes in the pig-human rearranged TADs.** A two side thypergeometric test were adopted to estimate the *P*-value.

File Name: Supplementary Data 12
Description: **The information of primer and genomic location of reporter assay.**
