# Supplementary material for: A compendium and comparative epigenomics analysis of cis-regulatory elements in the pig genome
Source: Nat Commun. 2021 Apr 13;12:2217. doi: 10.1038/s41467-021-22448-x (PMC8044108; doi:10.1038/s41467-021-22448-x)
Supplement: Supplementary file 15 — Reporting Summary [file 41467_2021_22448_MOESM15_ESM.pdf]

nature research

Corresponding author(s): Shuhong Zhao, Xinyun Li

Last updated by author(s): Feb 25, 2021

Reporting Summary

Nature Research wishes to improve the reproducibility of the work that we publish. This form provides structure for consistency and transparency in reporting. For further information on Nature Research policies, see our Editorial Policies and the Editorial Policy Checklist.

Statistics

For all statistical analyses, confirm that the following items are present in the figure legend, table legend, main text, or Methods section.

n/a	Confirmed	
☐	☒	The exact sample size (*n*) for each experimental group/condition, given as a discrete number and unit of measurement
☐	☒	A statement on whether measurements were taken from distinct samples or whether the same sample was measured repeatedly
☐	☒	The statistical test(s) used AND whether they are one- or two-sided<br>*Only common tests should be described solely by name; describe more complex techniques in the Methods section.*
☐	☒	A description of all covariates tested
☐	☒	A description of any assumptions or corrections, such as tests of normality and adjustment for multiple comparisons
☐	☒	A full description of the statistical parameters including central tendency (e.g. means) or other basic estimates (e.g. regression coefficient) AND variation (e.g. standard deviation) or associated estimates of uncertainty (e.g. confidence intervals)
☐	☒	For null hypothesis testing, the test statistic (e.g. *F*, *t*, *r*) with confidence intervals, effect sizes, degrees of freedom and *P* value noted<br>*Give P values as exact values whenever suitable.*
☒	☐	For Bayesian analysis, information on the choice of priors and Markov chain Monte Carlo settings
☒	☐	For hierarchical and complex designs, identification of the appropriate level for tests and full reporting of outcomes
☒	☐	Estimates of effect sizes (e.g. Cohen's *d*, Pearson's *r*), indicating how they were calculated

Our web collection on statistics for biologists contains articles on many of the points above.

Software and code

Policy information about availability of computer code

Data collection

Images processing from sequencing data using standard illumina Hiseq X ten PE150 pipeline (RNA-seq, ChIP-seq, ATAC-seq and Hi-C) and whole genome sequencing data which were obtained from NCBI databases.

Data analysis

Alignment and assembly of RNA-seq data were performed using TopHat algorithm (http://ccb.jhu.edu/software/tophat) and Cufflinks algorithm (http://cole-trapnell-lab.github.io/cufflinks/). Newly identification of lncRNAs and other types of transcripts were indicated in the manuscript's Methods section.
ChIP-seq data and ATAC-seq data were analyzed based on ENCODE ChIP-seq pipeline (https://github.com/kundajelab/chipseq_pipeline) and ATAC-seq pipeline (https://github.com/kundajelab/atac_dnase_pipelines), respectively.
Hi-C data analysis: Read mapping and matrix generation were conducted using HiC-Pro pipeline (https://github.com/nservant/HiC-Pro). The visualized contact matrix was generated using juicer system (https://github.com/aidenlab/juicer). HOMER program (http://homer.ucsd.edu/homer) was used to identify A/B compartment and create a normalized interaction matrix. The TAD structure (insulation/boundaries) was defined using perl cworld::dekker module (https://github.com/dekkerlab/cworld-dekker). Chromatin loops were found using HiCCUPS algorithm (https://github.com/aidenlab/juicer/wiki/HiCCUPS).
Short variants were obtained by employing GATK variant calling pipelines (https://software.broadinstitute.org/gatk).

The following programs and softwares were used:
TopHat (v2.1.1)
Cufflinks (v2.2.1)
CNCI (v2.0)
CPC (v2.0)
RSEM (v1.3.0)
deepTools (v2.0)

BLASTX (v.2.6.0)
BWA (v0.7.15)
SAMTools (v1.9)
MACS2 (v2.1.0)
ROSE (http://younglab.wi.mit.edu/super_enhancer_code.html)
BEDTools (v2.26.0)
LiftOver (https://genome.ucsc.edu/cgi-bin/hgLiftOver)
GREAT (v3.0.0)
HOMER (v4.10.3)
TOMTOM (v4.11.2)
Bowtie2 (v2.3.4.1)
Picard (v1.126)
TOBIAS (v0.10.1)
HiC-Pro (v2.9.0)
juicer (v1.8.9)
IGV (v2.4.18)
Treeview3 (https://bitbucket.org/TreeView3Dev/treeview3/)
TopDom (v0.0.2)
VCFtools (v0.1.15)
ToppGene (https://toppgene.cchmc.org/enrichment.jsp)
R (v3.4.3)
preprocessCore R package (v1.40.0)
pheatmap R package (v1.0.12)
DEseq2 R package (v1.16.1)
edgeR R package (v3.20.9)
R (v3.6.3)
qsmooth R package (v1.2.0)

For manuscripts utilizing custom algorithms or software that are central to the research but not yet described in published literature, software must be made available to editors and reviewers. We strongly encourage code deposition in a community repository (e.g. GitHub). See the Nature Research guidelines for submitting code & software for further information.

Data

Policy information about availability of data

All manuscripts must include a data availability statement. This statement should provide the following information, where applicable:

- Accession codes, unique identifiers, or web links for publicly available datasets
- A list of figures that have associated raw data
- A description of any restrictions on data availability

Raw sequencing data have been submitted to Sequence Read Archive (SRA) under the Bioproject accession number PRJNA597497. Processing data files have been submitted to Gene Expression Omnibus (GEO) under the accession number GSE143288.

Field-specific reporting

Please select the one below that is the best fit for your research. If you are not sure, read the appropriate sections before making your selection.

- [x] Life sciences
- [ ] Behavioural & social sciences
- [ ] Ecological, evolutionary & environmental sciences

For a reference copy of the document with all sections, see nature.com/documents/nr-reporting-summary-flat.pdf

Life sciences study design

All studies must disclose on these points even when the disclosure is negative.

Sample size	In our study, we collected a total of 61 samples of 12 tissues from two-week old piglets of two Western commercial (Large white and Duroc) and two Chinese local (Meishan and Enshi black) breeds. There was no statistical method was used to predetermine sample size.
Data exclusions	No data exclusion.
Replication	Two replicates were used for ChIP-seq, ATAC-seq, and RNA-seq. Four replicates were used for the Dual-Luciferase Reporter Assay.
Randomization	The predicted promoters and enhancers were verified by Dual-Luciferase Reporter Assay System, which were randomly selected.
Blinding	Blinding was not relevant to our study.

Reporting for specific materials, systems and methods

We require information from authors about some types of materials, experimental systems and methods used in many studies. Here, indicate whether each material, system or method listed is relevant to your study. If you are not sure if a list item applies to your research, read the appropriate section before selecting a response.

Materials & experimental systems

n/a	Involved in the study
☐	☒ Antibodies
☐	☒ Eukaryotic cell lines
☒	☐ Palaeontology and archaeology
☐	☒ Animals and other organisms
☒	☐ Human research participants
☒	☐ Clinical data
☒	☐ Dual use research of concern

Methods

n/a	Involved in the study
☐	☒ ChIP-seq
☒	☐ Flow cytometry
☒	☐ MRI-based neuroimaging

Antibodies

Antibodies used	H3K4me3 (Millipore Cat# 04-745, RRID: AB_1163444)<br>H3K27ac (Abcam Cat# ab4729, RRID: AB_2118291)
Validation	These two primary antibodies used are all high-quality commercial antibodies against Histone H3 modifications, validated as ChIP grade by the manufacturer (Millipore and Abcam):<br>https://www.emdmillipore.com/US/en/product/Anti-trimethyl-Histone-H3-Lys4-Antibody-clone-MC315-rabbit-monoclonal,MM_NF-04-745<br>https://www.abcam.com/histone-h3-acetyl-k27-antibody-chip-grade-ab4729.html<br>the Histone Antibody Specificity Database (http://www.histoneantibodies.com)<br>the Antibody Validation Database (http://compbio.med.harvard.edu/antibodies/).

Eukaryotic cell lines

Policy information about cell lines

Cell line source(s)	C2C12, 3D4-21, and HEK-293T cells were provided by ATCC.
Authentication	They were validated by the manufacturer (ATCC).
Mycoplasma contamination	They were tested negative for Mycoplasma by Myco-Blue Mycoplasma Detector kit (Vazyme, D101-01)
Commonly misidentified lines<br>(See ICLAC register)	No commonly misidentified cell lines were used in this study.

Animals and other organisms

Policy information about studies involving animals; ARRIVE guidelines recommended for reporting animal research

Laboratory animals	Four domestic pig breeds were selected, including two Chinese local breeds of Meishan (MS) and Enshi Black (ES) and two Western commercial breeds of Duroc and Large White (LW). The following tissue samples were collected from two male piglets at the age of two weeks from each breed: skeletal muscle, spleen, heart, kidney, liver, fat, lung, pancreas, thymus, cerebrum, cerebellum, and duodenum.
Wild animals	The study did not involve wild animals.
Field-collected samples	All sample were collected in Hubei Province P.R.China.
Ethics oversight	All experimental protocols were approved by the Ethics Committee of Huazhong Agricultural University (HZAUSW-2018-008).

Note that full information on the approval of the study protocol must also be provided in the manuscript.

ChIP-seq

Data deposition

☒ Confirm that both raw and final processed data have been deposited in a public database such as GEO.

☒ Confirm that you have deposited or provided access to graph files (e.g. BED files) for the called peaks.

Data access links<br>*May remain private before publication.*	https://www.ncbi.nlm.nih.gov/geo/query/acc.cgi?acc=GSE143288 (secure token: wzolycuephsrrcj)
Files in database submission	GSM4256252 Duroc_2W_1_Fat_H3K27ac

GSM4256253 Duroc_2W_2_Fat_H3K27ac
GSM4256254 Duroc_2W_1_Fat_H3K4me3
GSM4256255 Duroc_2W_2_Fat_H3K4me3
GSM4256256 Duroc_2W_1_Heart_H3K27ac
GSM4256257 Duroc_2W_2_Heart_H3K27ac
GSM4256258 Duroc_2W_1_Heart_H3K4me3
GSM4256259 Duroc_2W_2_Heart_H3K4me3
GSM4256260 Duroc_2W_1_Liver_H3K27ac
GSM4256261 Duroc_2W_2_Liver_H3K27ac
GSM4256262 Duroc_2W_1_Liver_H3K4me3
GSM4256263 Duroc_2W_2_Liver_H3K4me3
GSM4256264 Duroc_2W_1_Muscle_H3K27ac
GSM4256265 Duroc_2W_2_Muscle_H3K27ac
GSM4256266 Duroc_2W_1_Muscle_H3K4me3
GSM4256267 Duroc_2W_2_Muscle_H3K4me3
GSM4256268 Duroc_2W_1_Spleen_H3K27ac
GSM4256269 Duroc_2W_2_Spleen_H3K27ac
GSM4256270 Duroc_2W_1_Spleen_H3K4me3
GSM4256271 Duroc_2W_2_Spleen_H3K4me3
GSM4256272 ES_2W_1_Cerebellum_H3K27ac
GSM4256273 ES_2W_1_Cerebellum_H3K4me3
GSM4256274 ES_2W_1_Cerebrum_H3K27ac
GSM4256275 ES_2W_1_Cerebrum_H3K4me3
GSM4256276 ES_2W_1_Duodenum_H3K27ac
GSM4256277 ES_2W_1_Duodenum_H3K4me3
GSM4256278 ES_2W_1_Fat_H3K27ac
GSM4256279 ES_2W_2_Fat_H3K27ac
GSM4256280 ES_2W_1_Fat_H3K4me3
GSM4256281 ES_2W_2_Fat_H3K4me3
GSM4256282 ES_2W_1_Heart_H3K27ac
GSM4256283 ES_2W_2_Heart_H3K27ac
GSM4256284 ES_2W_1_Heart_H3K4me3
GSM4256285 ES_2W_2_Heart_H3K4me3
GSM4256286 ES_2W_1_Kidney_H3K27ac
GSM4256287 ES_2W_1_Kidney_H3K4me3
GSM4256288 ES_2W_1_Liver_H3K27ac
GSM4256289 ES_2W_2_Liver_H3K27ac
GSM4256290 ES_2W_1_Liver_H3K4me3
GSM4256291 ES_2W_2_Liver_H3K4me3
GSM4256292 ES_2W_1_Lung_H3K27ac
GSM4256293 ES_2W_1_Lung_H3K4me3
GSM4256294 ES_2W_1_Muscle_H3K27ac
GSM4256295 ES_2W_2_Muscle_H3K27ac
GSM4256296 ES_2W_1_Muscle_H3K4me3
GSM4256297 ES_2W_2_Muscle_H3K4me3
GSM4256298 ES_2W_1_Pancreas_H3K27ac
GSM4256299 ES_2W_1_Pancreas_H3K4me3
GSM4256300 ES_2W_1_Spleen_H3K27ac
GSM4256301 ES_2W_2_Spleen_H3K27ac
GSM4256302 ES_2W_1_Spleen_H3K4me3
GSM4256303 ES_2W_2_Spleen_H3K4me3
GSM4256304 ES_2W_1_Thymus_H3K27ac
GSM4256305 ES_2W_1_Thymus_H3K4me3
GSM4256306 LW_2W_1_Cerebellum_H3K27ac
GSM4256307 LW_2W_2_Cerebellum_H3K27ac
GSM4256308 LW_2W_1_Cerebellum_H3K4me3
GSM4256309 LW_2W_2_Cerebellum_H3K4me3
GSM4256310 LW_2W_1_Cerebrum_H3K27ac
GSM4256311 LW_2W_2_Cerebrum_H3K27ac
GSM4256312 LW_2W_1_Cerebrum_H3K4me3
GSM4256313 LW_2W_2_Cerebrum_H3K4me3
GSM4256314 LW_2W_1_Duodenum_H3K27ac
GSM4256315 LW_2W_2_Duodenum_H3K27ac
GSM4256316 LW_2W_1_Duodenum_H3K4me3
GSM4256317 LW_2W_2_Duodenum_H3K4me3
GSM4256318 LW_2W_1_Fat_H3K27ac
GSM4256319 LW_2W_2_Fat_H3K27ac
GSM4256320 LW_2W_1_Fat_H3K4me3

GSM4256321 LW_2W_2_Fat_H3K4me3
GSM4256322 LW_2W_1_Heart_H3K27ac
GSM4256323 LW_2W_2_Heart_H3K27ac
GSM4256324 LW_2W_1_Heart_H3K4me3
GSM4256325 LW_2W_2_Heart_H3K4me3
GSM4256326 LW_2W_1_Kidney_H3K27ac
GSM4256327 LW_2W_2_Kidney_H3K27ac
GSM4256328 LW_2W_1_Kidney_H3K4me3
GSM4256329 LW_2W_2_Kidney_H3K4me3
GSM4256330 LW_2W_1_Liver_H3K27ac
GSM4256331 LW_2W_2_Liver_H3K27ac
GSM4256332 LW_2W_1_Liver_H3K4me3
GSM4256333 LW_2W_2_Liver_H3K4me3
GSM4256334 LW_2W_1_Lung_H3K27ac
GSM4256335 LW_2W_2_Lung_H3K27ac
GSM4256336 LW_2W_1_Lung_H3K4me3
GSM4256337 LW_2W_2_Lung_H3K4me3
GSM4256338 LW_2W_1_Muscle_H3K27ac
GSM4256339 LW_2W_2_Muscle_H3K27ac
GSM4256340 LW_2W_1_Muscle_H3K4me3
GSM4256341 LW_2W_2_Muscle_H3K4me3
GSM4256342 LW_2W_1_Pancreas_H3K27ac
GSM4256343 LW_2W_2_Pancreas_H3K27ac
GSM4256344 LW_2W_1_Pancreas_H3K4me3
GSM4256345 LW_2W_2_Pancreas_H3K4me3
GSM4256346 LW_2W_1_Spleen_H3K27ac
GSM4256347 LW_2W_2_Spleen_H3K27ac
GSM4256348 LW_2W_1_Spleen_H3K4me3
GSM4256349 LW_2W_2_Spleen_H3K4me3
GSM4256350 LW_2W_1_Thymus_H3K27ac
GSM4256351 LW_2W_2_Thymus_H3K27ac
GSM4256352 LW_2W_1_Thymus_H3K4me3
GSM4256353 LW_2W_2_Thymus_H3K4me3
GSM4256354 MS_2W_1_Fat_H3K27ac
GSM4256355 MS_2W_2_Fat_H3K27ac
GSM4256356 MS_2W_1_Fat_H3K4me3
GSM4256357 MS_2W_2_Fat_H3K4me3
GSM4256358 MS_2W_1_Heart_H3K27ac
GSM4256359 MS_2W_2_Heart_H3K27ac
GSM4256360 MS_2W_1_Heart_H3K4me3
GSM4256361 MS_2W_2_Heart_H3K4me3
GSM4256362 MS_2W_1_Liver_H3K27ac
GSM4256363 MS_2W_2_Liver_H3K27ac
GSM4256364 MS_2W_1_Liver_H3K4me3
GSM4256365 MS_2W_2_Liver_H3K4me3
GSM4256366 MS_2W_1_Muscle_H3K27ac
GSM4256367 MS_2W_2_Muscle_H3K27ac
GSM4256368 MS_2W_1_Muscle_H3K4me3
GSM4256369 MS_2W_2_Muscle_H3K4me3
GSM4256370 MS_2W_1_Spleen_H3K27ac

Genome browser session
(e.g. UCSC)

The genome browser tracks were loaded to Integrative Genomics Viewer (IGV v2.4.18) (http://software.broadinstitute.org/software/igv/) to view signal.

Methodology

Replicates

Most of tissues for each breed contain two biological replicates as described in Supplementary Table .

Sequencing depth

Read length:150bp
Sequencing type: paired-end.
The filtered reads were presented in Supplementary Data 1.

Antibodies

H3K4me3 (Millipore Cat# 04-745, RRID: AB_1163444)
H3K27ac (Abcam Cat# ab4729, RRID: AB_2118291)

Peak calling parameters	Narrow peaks were generated with MACS2 v2.1.0 (-g genome.size -p 0.01 --nomodel --shift 0 --extsize n --keep-dup 1 -B --SPMR). Broad peaks were detected with MACS2 v2.1.0 (-g genome.size -p 0.01 --nomodel --shift 0 --extsize n --keep-dup 1 --broad). The "genome.size" was the sum calculated from the chromosome size files downloaded from the UCSC browser, which were named as "susScr11.chrom.sizes" or "susScr3.chrom.sizes". The "n" for the extsize parameter was calculated automatically by the ENCODE ChIP-seq pipeline, which was downloaded from GitHub website (https://github.com/kundajelab/chipseq_pipeline).
Data quality	Read coverage of genomic regions for filtered BAM files was computed to assess the genome-wide similarities of replicate BAM files with 2 kb bin size by multiBamSummary bins function in deepTools v2.0. If the Pearson correlation coefficients of read coverage were >0.83, the replicate BAM files were merged as one ChIP BAM file or Input BAM file for peak calling. Peaks were further processed by following four steps: (i) narrow peaks with P>0.00001 were filtered out; (ii) read coverage of 2kb region centered at the midpoint of peaks was calculated with multiBamSummary BED-file function in deepTools v2.0 and normalized with read depth : IPrpm=IP(peak region total reads)/IP(total mapped reads per million) and INPUTrpm=INPUT(peak region total reads)/INPUT (total mapped reads per million); (iii) the enriched regions for H3K4me3 and H3K27ac were defined with more than a two-fold change of normalized read coverage (IPrpm>2×INPUTrpm) and with normalized read coverage change (IPrpm-INPUTrpm)>1; and (iv) the overlap of enriched regions were merged, and then the 2 kb regions centered at the midpoint of merged regions were acquired to identify enhancers and promoters.
Software	ChIP-seq datasets were processed with reference to the ENCODE ChIP-seq pipeline (https://github.com/kundajelab/chipseq_pipeline). The ChIP reads and Input reads were aligned to susScr11 and susScr3 genome assemblies using BWA v0.7.15. The filtered BAM files were generated by removing low MAPQ reads (<25), unmapped reads, mate unmapped reads, not primary alignments, reads failing platform, and duplicates using SAMTools v1.9. and Picard v1.126 (https://broadinstitute.github.io/picard).
