# Supplementary material for: A compendium and comparative epigenomics analysis of cis-regulatory elements in the pig genome
Source: Nat Commun. 2021 Apr 13;12:2217. doi: 10.1038/s41467-021-22448-x (PMC8044108; doi:10.1038/s41467-021-22448-x)
Supplement: Supplementary file 16 — Source Data [file 41467_2021_22448_MOESM16_ESM.pptx]

## Slide 1
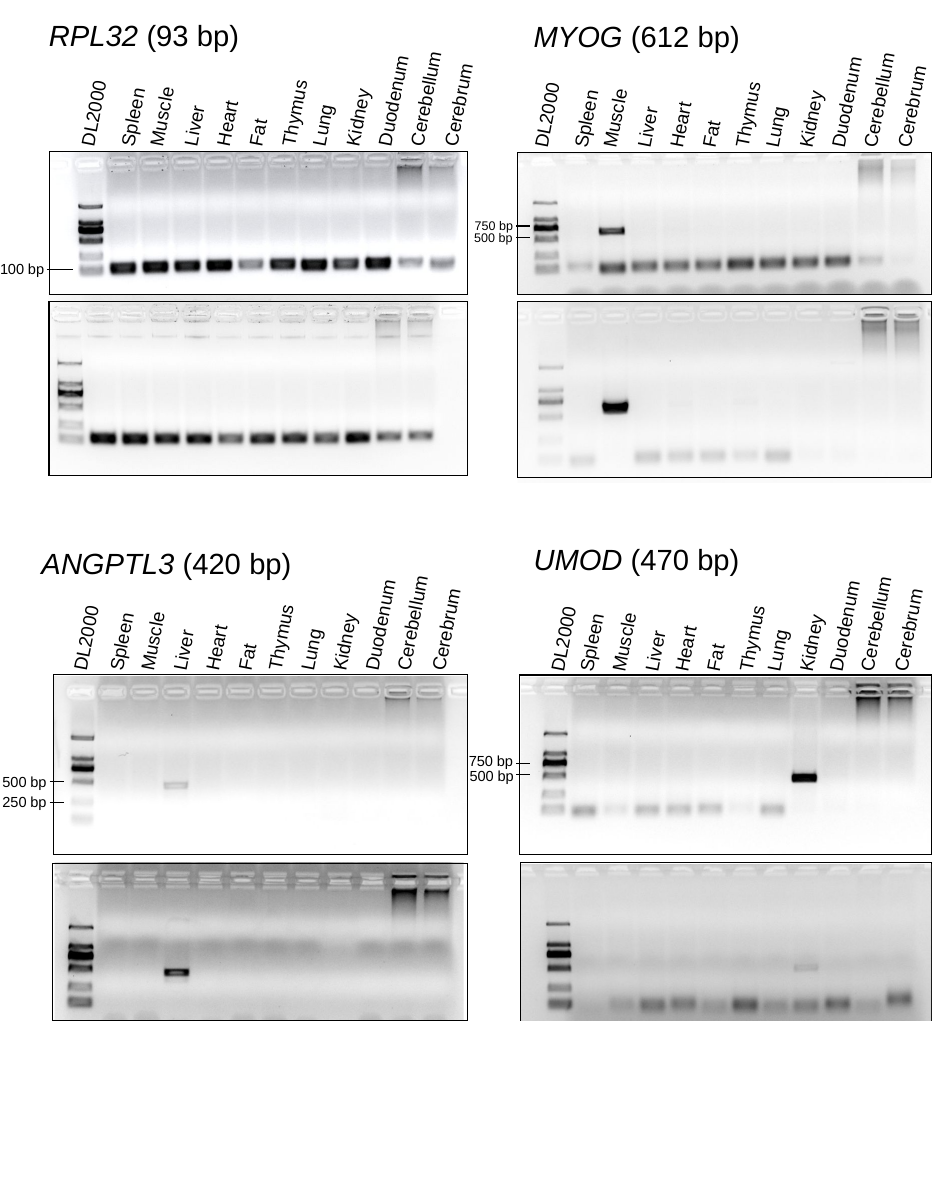

RPL32 (93 bp)
# MYOG (612 bp)
Cerebrum
Cerebellum
Cerebrum
Cerebellum
Duodenum
Duodenum
DL2000
Spleen
Muscle
Liver
Heart
Fat
Thymus
Lung
Kidney
DL2000
Spleen
Muscle
Liver
Heart
Fat
Thymus
Lung
Kidney
750 bp
500 bp
100 bp
UMOD (470 bp)
ANGPTL3 (420 bp)
Cerebrum
Cerebellum
Cerebrum
Cerebellum
Duodenum
Duodenum
DL2000
Spleen
Muscle
Liver
Heart
Fat
Thymus
Lung
Kidney
DL2000
Spleen
Muscle
Liver
Heart
Fat
Thymus
Lung
Kidney
750 bp
500 bp
500 bp
250 bp

## Slide 2
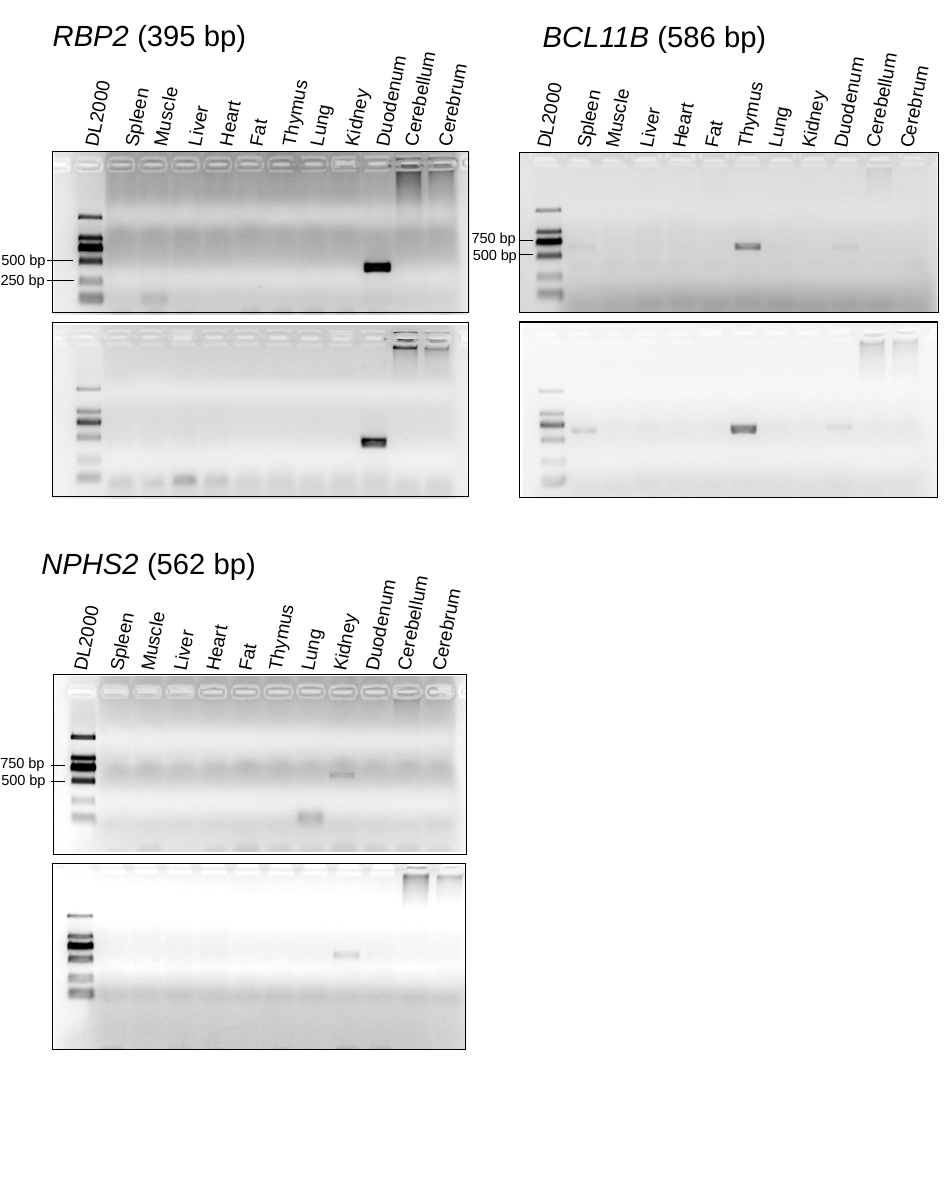

RBP2 (395 bp)
# BCL11B (586 bp)
Cerebrum
Cerebellum
Cerebrum
Cerebellum
Duodenum
Duodenum
DL2000
Spleen
Muscle
Liver
Heart
Fat
Thymus
Lung
Kidney
DL2000
Spleen
Muscle
Liver
Heart
Fat
Thymus
Lung
Kidney
750 bp
500 bp
500 bp
250 bp
NPHS2 (562 bp)
Cerebrum
Cerebellum
Duodenum
DL2000
Spleen
Muscle
Liver
Heart
Fat
Thymus
Lung
Kidney
750 bp
500 bp
